# Magnesium-binding architectures in RNA crystal structures: validation, binding preferences, classification and motif detection

**DOI:** 10.1093/nar/gkv225

**Published:** 2015-03-23

**Authors:** Heping Zheng, Ivan G. Shabalin, Katarzyna B. Handing, Janusz M. Bujnicki, Wladek Minor

**Affiliations:** 1 Department of Molecular Physiology and Biological Physics, University of Virginia, Charlottesville, VA 22908-0736, USA; 2 Center for Structural Genomics of Infectious Diseases (CSGID) Consortium, USA; 3 Midwest Center for Structural Genomics (MCSG) Consortium, USA; 4 New York Structural Genomics Research Consortium (NYSGRC), USA; 5 Enzyme Function Initiative (EFI), USA; 6 Laboratory of Bioinformatics and Protein Engineering, International Institute of Molecular and Cell Biology in Warsaw, Warsaw 02-109, Poland; 7 Institute of Molecular Biology and Biotechnology, Faculty of Biology, Adam Mickiewicz University, Umultowska 89, 61-614 Poznan, Poland

## Abstract

The ubiquitous presence of magnesium ions in RNA has long been recognized as a key factor governing RNA folding, and is crucial for many diverse functions of RNA molecules. In this work, Mg^2+^-binding architectures in RNA were systematically studied using a database of RNA crystal structures from the Protein Data Bank (PDB). Due to the abundance of poorly modeled or incorrectly identified Mg^2+^ ions, the set of all sites was comprehensively validated and filtered to identify a benchmark dataset of 15 334 ‘reliable’ RNA-bound Mg^2+^ sites. The normalized frequencies by which specific RNA atoms coordinate Mg^2+^ were derived for both the inner and outer coordination spheres. A hierarchical classification system of Mg^2+^ sites in RNA structures was designed and applied to the benchmark dataset, yielding a set of 41 types of inner-sphere and 95 types of outer-sphere coordinating patterns. This classification system has also been applied to describe six previously reported Mg^2+^-binding motifs and detect them in new RNA structures. Investigation of the most populous site types resulted in the identification of seven novel Mg^2+^-binding motifs, and all RNA structures in the PDB were screened for the presence of these motifs.

## INTRODUCTION

Metal ions are indispensable for proper RNA folding, stability and function in various biological processes (1). The positive charge of metal cations is needed to compensate for the negative charge of RNA's highly acidic phosphate backbone, permitting RNA to form and retain compact and specific three-dimensional structures (2). The resulting structural complexity and wide repertoire of structural arrangements allows RNA to effectively perform a multitude of key cellular functions. In addition to their ubiquitous role as counter ions, metal ions are also crucial for some RNA molecules to recognize binding partners (3,4). In some ribozymes, metal ions have been found to directly mediate catalytic processes (5).

Mg^2+^ is generally accepted as the most important ion for RNA stabilization (1,6) and is the most frequently identified metal in RNA structures. Magnesium ions are nearly ubiquitous in RNA structures and many different types of coordination architectures have been observed for Mg^2+^ in RNA. A comprehensive survey of Mg^2+^ binding sites in RNA should be particularly useful for the prediction and annotation of RNA structure, function and the recognition of binding partners. Recent advancements in macromolecule crystallography have led to the determination of many structurally diverse metal-containing RNA crystal structures, offering a unique opportunity for such a survey of Mg^2+^ binding sites.

Most previous studies of Mg^2+^-binding architectures in RNA were not performed on a variety of crystal structures of different RNA families but were limited to the analysis of a single structure (2,7–12). However, two databases specialized for the investigation of metal ions in multiple RNA structures are available: MeRNA (13) and MINAS (14). MeRNA focuses on eight previously reported metal-binding motifs and is based on 389 structures deposited in the Protein Data Bank (PDB) (15) before February 2007. MINAS offers a multitude of search functions for metal ligands defined by element, functional group or residue through all RNA structures in the PDB. Nevertheless, neither MeRNA nor MINAS offers a readily interpretable, systematic classification of Mg^2+^ in RNA structures.

A few classification schemes of metal ion binding sites in RNA crystal structures have been proposed (10,11). Based on the analysis of one large ribosomal subunit, Klein *et al*. provided a simplified model to classify magnesium binding sites into six types based on the number and geometric isomerism of non-water ligands in the inner sphere (10). Lippert *et al*. evaluated possible inner-sphere interactions by phosphate group, sugar entity, nucleobase and their theoretical combinations and generalized more than 50 binding patterns of a metal ion by a single nucleotide (11). Unfortunately, outer-sphere ligands were not described systematically in either of these studies. Yet in many cases the inner sphere around a magnesium ion is composed of only (or mostly) water molecules, and the specific structural features of these sites can only be differentiated by outer-sphere interactions with RNA. Additionally, no comprehensive analysis has been performed on the likelihood of different nucleotides to bind Mg^2+^ or on the relative abundance of each particular Mg^2+^-binding architecture.

A potential pitfall in classifying and surveying Mg^2+^ binding sites is the widespread misidentification of Mg^2+^ in RNA crystal structures. The misinterpretation of small molecules and ions bound to macromolecules, including metals, has been spotted in many macromolecule structures (16,17) and Mg^2+^ is not an exception (18). Mg^2+^ has the same number of electrons as water and Na^+^, neither of which can be distinguished from Mg^2+^ by difference electron density maps alone (19). Moreover, the X-ray absorption K-edge (9.5 Å) of Mg^2+^ is outside of the wavelength range producible by a typical synchrotron beamline, which makes it difficult to localize and differentiate it from water or Na^+^ by anomalous scattering. A significant number of incorrectly identified metal sites can impose a strong bias on metal binding analysis, especially for large-scale studies where it is infeasible to manually analyze and verify each site individually. Therefore it is critical to retrieve only trustworthy sites (i.e. sites in agreement with experimental data and known bioinorganic chemistry) for analysis.

In the present study, a systematic analysis of Mg^2+^ binding by RNA was performed with the following five objectives: (i) validate Mg^2+^ binding sites in RNA crystal structures deposited to the PDB and discard poorly modeled or misidentified sites; (ii) statistically analyze preferences of nucleotides and their individual atoms for Mg^2+^ coordination; (iii) craft and apply a hierarchical Mg^2+^ site classification system which takes into account both inner- and outer-sphere ligands; (iv) employ the classification system for describing and detecting Mg^2+^-binding motifs in RNA structures and (v) discover new Mg^2+^-binding motifs within populous site types.

## MATERIALS AND METHODS

Terminology, abbreviations and definitions used in this manuscript are provided as Supplementary Lexicon 1.

### Dataset used for analysis

The initial screening set comprises all structures in the PDB (15) deposited on or before 30 September 2014 that satisfied three criteria: (i) determined by X-ray crystallography, (ii) contains at least three common ribonucleotide residues (A, G, C or U) covalently linked by phosphodiester bonds and (iii) contains at least one modeled Mg^2+^ ion. This dataset of all Mg^2+^ sites is herein referred to as the ‘full dataset.’ Mg^2+^ sites were analyzed using the *NEIGHBORHOOD* database (20), which takes into account crystallographic symmetry and stores information on all identified atoms and residues together with their interactions with neighboring atoms and residues. Structures containing at least one ribosomal subunit were assigned to the ‘ribosome’ subset, while all other structures were placed in the ‘non-ribosome’ subset.

### Types of coordinating atoms from ribonucleotides

The atoms from common ribonucleotides that can potentially coordinate Mg^2+^ were classified into four different types: (i) O_ph_, phosphate oxygen (OP1/OP2); (ii) O_r_, oxygens in ribose (O2′/O4′) or oxygens bridging phosphate and ribose (O3′/O5′); (iii) O_b_, nucleobase oxygen and (iv) N_b_, nucleobase nitrogen.

### Definition of inner-sphere ligand atoms and coordination number

Only oxygen and nitrogen were considered as potential inner-sphere ligand atoms. The search for inner-sphere ligand atoms was performed in two steps to optimally account for the potentially large metal-ligand distance deviations in RNA structures determined at medium to low resolution. The ideal distances (*d*_ideal_) for Mg^2+^−O (2.08 Å) and Mg^2+^−N (2.20 Å) bonds were defined as the mean distances observed in the Cambridge Structural Database (21). In the first step, all oxygen and nitrogen atoms with a distance *d* to a magnesium ion where *d* ≤ *d*_ideal_ + 0.5 Å were identified as inner-sphere ligand atoms.

The second step, which was performed only if the number of ligand atoms found in the first step was less than six, included ligands with distances up to 0.5 Å longer (*d*_ideal_+0.5 Å < *d* ≤ *d*_ideal_+1.0 Å). This second step identified additional nearby oxygen and nitrogen atoms which could potentially complete the octahedral geometry of the inner sphere. Three additional rules were applied in this second step to preclude chemically unfavorable interactions: (i) a second oxygen from the same phosphate group was not accepted since a phosphate group cannot form a bidentate interaction with Mg^2+^ (22); (ii) the only allowed coordinating nitrogen atoms from a nucleobase were endocyclic nitrogens with a lone electron pair in the plane of the aromatic ring (–N = ) and (iii) ligand atoms were accepted only if they have a planar ligand-Mg-ligand angle greater than 50º with all previously found ligands.

The coordination number (CN) of a magnesium ion was defined as the number of inner-sphere ligand atoms identified by the procedure outlined above.

### Definition of outer-sphere atoms

Outer-sphere coordinating atoms were identified based on the presence of hydrogen bonds to any inner-sphere water molecules. Hydrogen bonds were identified by the *Probe* program (23) from the *MolProbity* suite (24). For crystallographic symmetry related interactions, hydrogen bonds were identified by the *CONTACT* program from the CCP4 suite (25).

### Outer-sphere moieties

For annotation of outer-sphere interactions, each individual RNA moiety (phosphate, ribose or nucleobase) was counted only once, and only if it did not form inner-sphere interactions with a given Mg^2+^ ion. If two or more moieties contribute hydrogen bonds to the same water molecule, these were still counted as separate moieties. O3′ and O5′ atoms were assigned to the ribose moiety; except when the connected phosphate moiety contributes to the inner or outer sphere of the same magnesium ion, the O3′ and O5′ atoms were considered to be part of the phosphate group. Outer-sphere moieties were labeled as P_out_ (phosphate), R_out_ (ribose) and B_out_ (nucleobase).

### Validation parameters

Three customized parameters *Q_v_, Q_s_* and *Q_e_* were used to quantitatively identify the ‘quality’ of Mg^2+^sites (in addition to other criteria). The value of each parameter has a maximum value of 1, with a lower value indicating poorer reliability and a higher value indicating better reliability.

*Q_v_* measures the agreement of the bond valence summation ($\sum {V_i }$) of the inner-sphere interactions with the oxidation state of magnesium (+2) as defined by $Q_v = 1 - \frac{{|\sum {V_i - 2} |}}{2}$, in which *V_i_* is the bond valence value derived from Mg^2+^-ligand distance of coordinating ligand *i* (26). A table of the relationships between distance and valence is provided for both Mg^2+^−O and Mg^2+^−N separately (Supplementary Table S1).

*Q_s_* measures the geometrical symmetry of the ligands distribution around the Mg^2+^ required for octahedral geometry by calculating the amplitude of the vector sum of the bond valence vectors **v**_i_ (27). The sum should be of magnitude 0 for a perfectly symmetrical set of ligands. *Q_s_* is defined as $Q_s = \frac{{|{\bf v}_1 + {\bf v}_2 + \cdots {\bf v}_n |}}{{\sum {V_i } }}$.

*Q_e_* measures the agreement of the isotropic atomic displacement parameter (B-factor) of the Mg^2+^ (*B_m_*) and its occupancy (*O_m_*) compared to those of all atoms in its environment (*B_e_, O_e_*). The environmental B-factor (*B_e_*) and occupancy (*O_e_*) were calculated as the valence weighted sum of those parameters for all non-hydrogen atoms within 4 Å of the Mg^2+^ divided by the overall valence ($B_e = \frac{{\sum {V_i B_i } }}{{\sum {V_i } }}$, $Q_e = \frac{{\sum {V_i O_i } }}{{\sum {V_i } }}$). In the majority of cases, both the Mg^2+^ and the atoms in its environment have full occupancy ($O_m = O_e = 1$) and *Q_e_* is defined as the smaller B-factor $\min (B_m ,B_e )$ divided by the larger B-factor $\max (B_m ,B_e )$. When partial occupancy was encountered for a magnesium ion or its environment, *Q_e_* was weighted by the occupancy using the formula:

\begin{equation*} \min(O_m, O_e)\times \min \left ( \frac{^{B_m}/_{O_m}}{^{B_e}/_{O_e}}, \frac{^{B_e}/_{O_e}}{^{B_m}/_{O_m}} \right ). \end{equation*}

### Normalized interaction frequencies of coordinating atoms and moieties

Normalized interaction frequencies of atoms (*F*_atom_) were calculated in a similar manner as has been reported previously (20). For an atom of type X, *F*_atom_(X) was calculated by the formula $F_{{\rm atom}} (X) = \frac{{p({\rm Mg - X})}}{{p({\rm X})}}$. *p*(Mg-X) is defined as the percentage of Mg-X interactions out of the total number of interactions for all coordinating atoms in the benchmark dataset. *p*(X) is defined as the percentage of atoms of type X out of all coordinating atoms in the full dataset. In other words, *F*_atom_ reflects the frequency that a certain type of atom is observed to coordinate Mg^2+^, normalized by the frequency of that type of atom in the full dataset. Hence, if *F*_atom_(X) > 1 and thus *p*(Mg-X) > *p*(X), atoms of type X interact with Mg^2+^ with relatively high frequency. Conversely, if *F*_atom_(X) < 1 and thus *p*(Mg-X) < *p*(X), Mg-X interactions occur with relatively low frequency.

### Geometric isomerism

Mg^2+^ sites coordinated more than one phosphate group (O_ph_) in the inner sphere were differentiated by geometrical arrangement as either *cis-* or *trans-* isoforms (two or four O_ph_) or *fac-* or *mer-* isoforms (three O_ph_). The *trans-* or *mer-* isoforms were defined by the presence of two O_ph_ atoms opposite to each other in a *trans-* conformation, defined as a ligand-Mg-ligand angle larger than 135º. In the absence of such pair of opposite O_ph_ ligands, the site was defined as *cis-* (two O_ph_) or *fac-* (three O_ph_).

## RESULTS

### Prevalence of poorly coordinated Mg^2+^ sites in RNA crystal structures from the PDB

**Figure 1.**
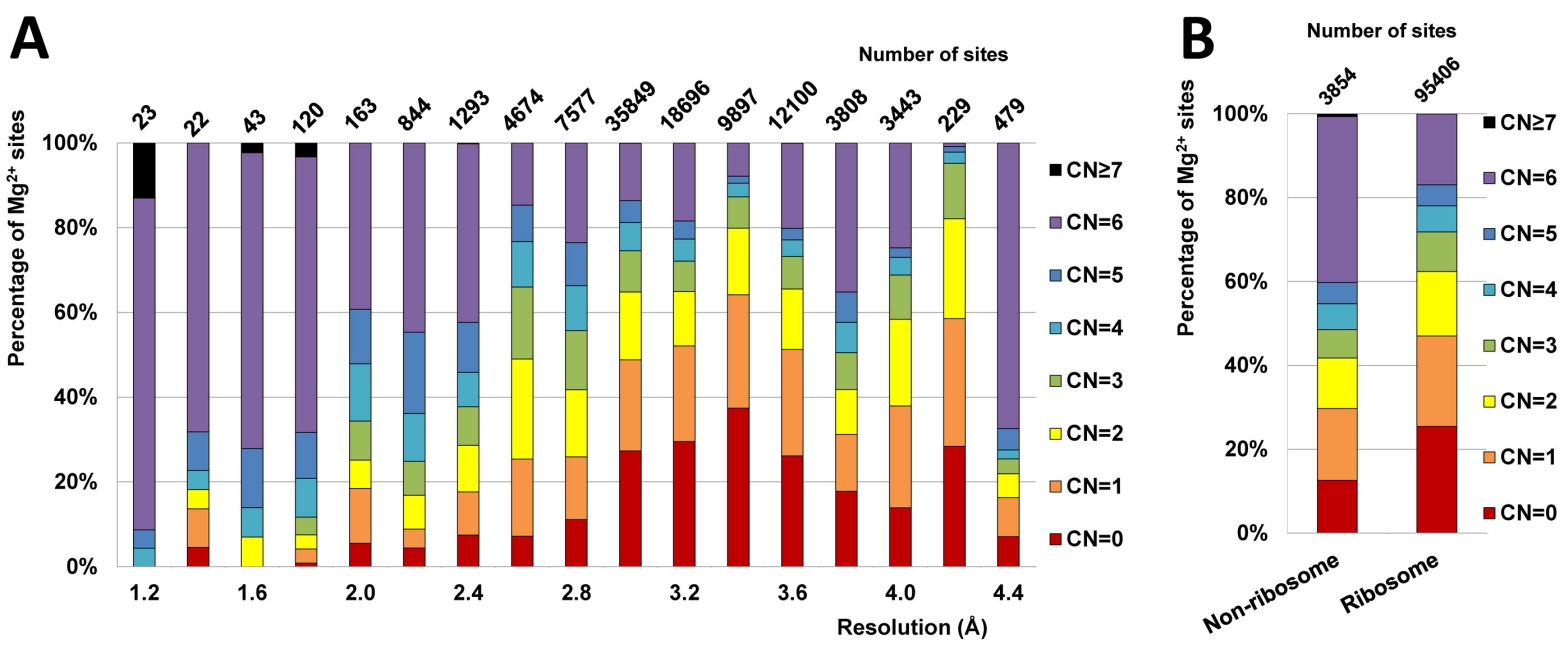
Statistics for the coordination number (CN) of Mg^2+^ sites in the full dataset. (**A**) Fraction of different CN for Mg^2+^ sites from structures in different resolution ranges shown as stacked bars. The total numbers of Mg^2+^ sites for each resolution range are shown on top of each bar. (**B**) Percentages of Mg^2+^ sites in either the ribosome or non-ribosome subset with a given CN.

**Figure 2.**
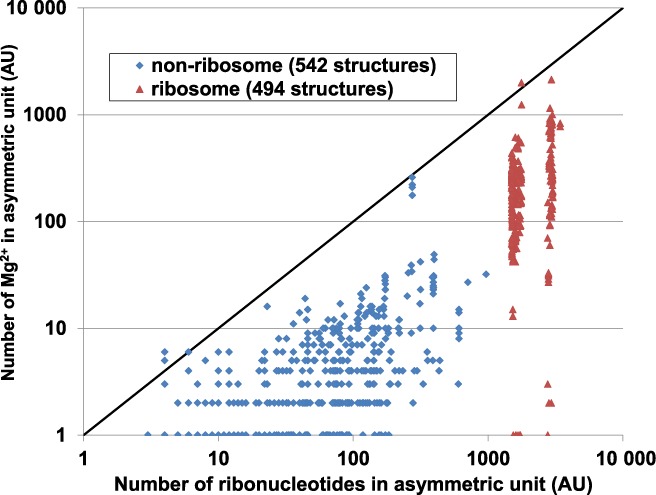
Number of Mg^2+^ ions versus number of ribonucleotides in the asymmetric unit (AU), as plotted separately for ribosome (red) and non-ribosome structures (blue) on a log–log plot. A line indicating an equal number of ribonucleotides and Mg^2+^ ions is shown. It must be noted that not all Mg^2+^ are modeled in X-ray crystal structures due to diffusely bound Mg^2+^, resolution limitations and model building strategies.

The full dataset contains 99260 Mg^2+^ sites from 1036 structures, consisting of 95406 sites from 494 ribosome structures and 3854 sites from 542 non-ribosome structures. Most sites in the full dataset are from low-resolution structures; only 2.5% of the sites (2508 sites) are from structures determined at a resolution better than 2.4 Å (Figure 1A). All structures are of resolution better than 4.5 Å, and most sites found in structures of resolution worse than 2.1 Å come from ribosome (Supplementary Figure S1). Most ribosomal structures in the full dataset, including both large subunit (∼3000 nucleotides) and small subunit (∼1500 nucleotides) structures, contain 100–1000 Mg^2+^, though a few contain five or fewer Mg^2+^ sites. Notably, 42% of the ribosome structures in the PDB do not contain a single Mg^2+^ site. The majority of the non-ribosome structures contain fewer than 10 Mg^2+^ sites each (Figure 2). This trend indicates that the number of modeled Mg^2+^ sites located by X-ray crystallography is often insufficient to neutralize the negative charge of RNA due to diffusely bound Mg^2+^, presence of other cations, limited resolution of the crystal structure and/or difficulty in Mg^2+^ identification.

More than half of the Mg^2+^ sites in the full dataset exhibit a highly incomplete inner sphere with coordination number (CN) in the range of 0–3 (Figure 1B), even when a very generous distance cutoff (1 Å above the ideal distance) is used in the search for inner-sphere atoms. Though sites in structures of higher resolution (≤2.0 Å) have a higher average CN, Mg^2+^ sites with a CN of three or less are still commonly observed (Figure 1A). In the full dataset, Mg^2+^ sites with a (relatively) complete inner sphere (CN = 4–6) compose around 30% of all sites in ribosome and around 50% of all sites in non-ribosome structures. A small number of sites (53, <0.1%) had CN>6. Manual inspection of the sites with CN>6 revealed modeling errors with severe clashes between the inner-sphere ligands and/or unlikely bidentate coordination by phosphate (22).

Mg^2+^ sites from structures determined at 2.9–3.7 Å resolution show the most incomplete inner spheres. For resolutions better than 2.9 Å, the completeness of coordination appears to be correlated with resolution (higher mean CN at better resolutions and lower mean CN at worse resolutions). This trend reverses at resolution worse than 3.7 Å, and well-coordinated sites become more abundant in worse resolution structures, likely due to the common practice of using a restrained hexahydrated Mg^2+^ during refinement instead of a single Mg^2+^ at lower resolutions with very poor electron density maps (28,29).

### Benchmark dataset

The benchmark dataset, which excludes highly questionable Mg^2+^ sites, was used for all further analyses. Mg^2+^ sites were included in the benchmark dataset only if all of the following criteria were met: (i) the site is RNA-bound (through inner and/or outer sphere); (ii) the site CN = 4–6; (iii) all three validation parameters are higher or equal to the threshold values (*Q_v_* ≥ 0.5, *Q_s_* ≥ 0.6 and *Q_e_* ≥ 0.5) as determined in the preliminary research described in the supplementary data (Supplementary Text 1, Supplementary Figures S2/S3); (iv) sites should not be coordinated by nucleobase nitrogen other than an endocyclic nitrogen with a lone electron pair in the plane of the aromatic ring (–N = ) in the inner sphere.

The benchmark dataset consists of 15334 Mg^2+^ binding sites (489 structures), which constitutes only 15% of the full dataset. The size of the benchmark dataset is significantly smaller than the size of the full dataset, mostly due to the abundance of sites with a highly incomplete Mg^2+^ inner sphere (CN = 0–3) in the full dataset (Figure 1). The majority of sites (80%) in the benchmark dataset have a complete coordination sphere (CN = 6), 15% of sites have CN = 5, and 5% of sites have CN = 4. The dataset comprises 14 682 sites from 294 ribosomal structures (15% of the original 95406 sites) and 652 sites from 195 non-ribosomal structures (17% of the original 3854 sites).

Coordination bond distances were investigated to verify the proper selection of reasonable sites for the benchmark dataset and to further investigate modeling problems found at Mg^2+^ sites (Supplementary Figure S4). The inner-sphere Mg^2+^−O (non-water) and Mg^2+^−N distance distributions show that more often than not, sites in the full dataset were modeled with these distances much longer than ideal values. Many of the sites in the full dataset that are observed with interaction distances longer than 2.4 Å might have been incorrectly identified as Mg^2+^ instead of water oxygen atoms, potassium ions or sodium ions, which have ideal bond distances to oxygen of 2.9 Å, 2.8 Å and 2.5 Å, respectively (27). Most of the potentially misidentified sites with distances much longer than ideal values were excluded from the benchmark dataset by the validation procedure described above (Supplementary Figure S4). A small number of interactions in the sites of the benchmark dataset are far from ideal, but those interactions are found within sites where most of the other inner-sphere interactions do not significantly deviate from ideal distance values.

### Frequencies of nucleotide atoms to coordinate magnesium ions

**Figure 3.**
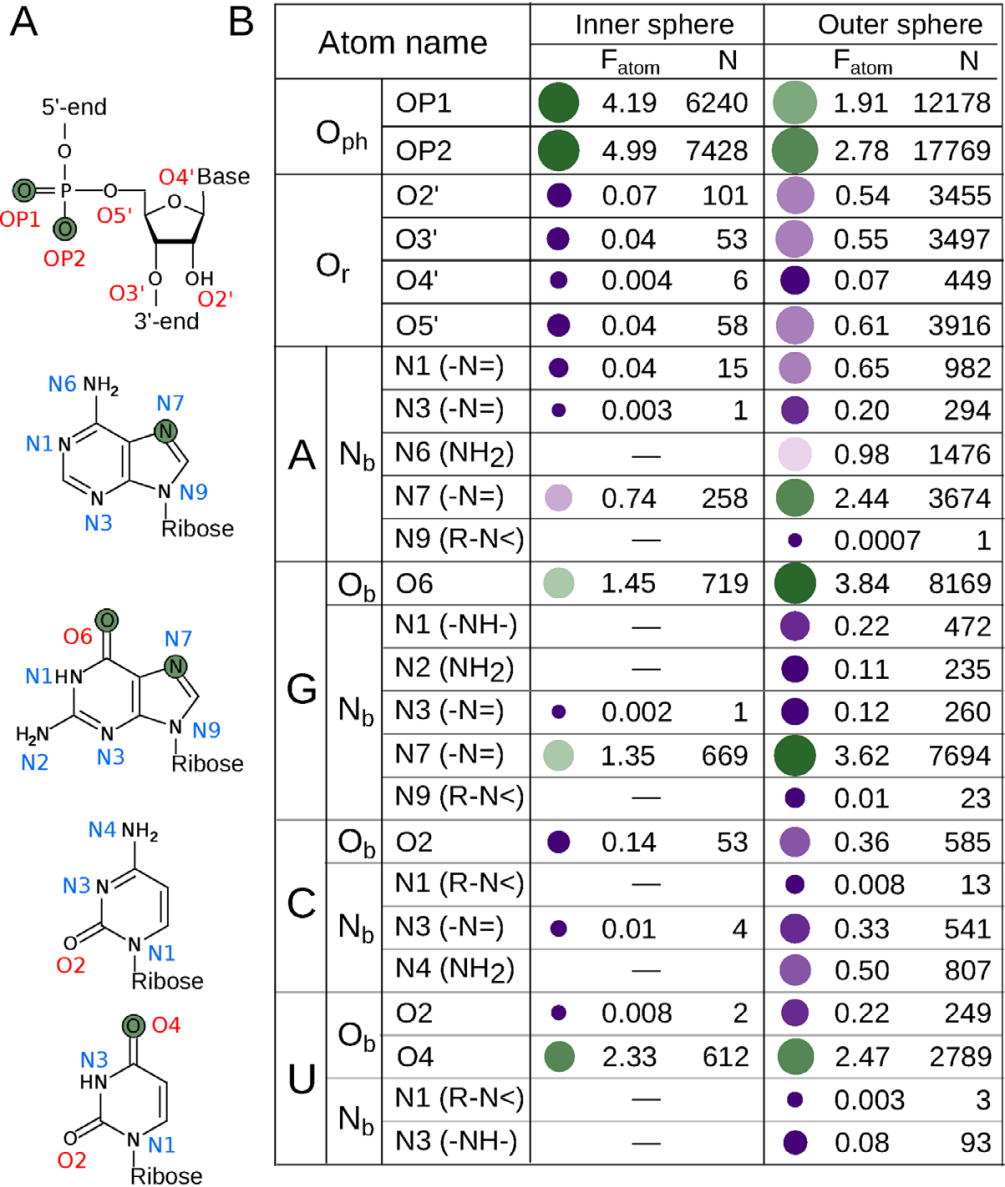
Normalized interaction frequencies of RNA atoms (*F*_atom_) for Mg^2+^ coordination in the inner sphere and outer sphere as calculated based on the benchmark dataset. (**A**) Chemical structures of nucleotides. The atoms with the highest *F*_atom_ values for Mg^2+^ coordination are colored in green. (**B**) *F*_atom_ values and the number of interactions (*N*) of RNA atoms with Mg^2+^. Dash for some of the nitrogen atoms represents chemically infeasible interactions for Mg^2+^ inner-sphere coordination which were excluded from the benchmark dataset by definition. Coordinating atoms with *F*_atom_ higher than one are indicated by green bubbles with more saturated colors representing higher values. Coordinating atoms with *F*_atom_ lower than one are indicated by magenta bubbles with more saturated colors representing lower values. The size of each bubble indirectly indicates the number of interactions.

The frequencies of RNA atoms to coordinate Mg^2+^ were evaluated using the normalized interaction frequency (*F*_atom_), which measures the frequency a particular kind of atom served as a ligand normalized by the frequency of that atom type in RNA structures overall. The most commonly observed inner-sphere ligand atom type is oxygen from phosphate (O_ph_) (Figure 3, Supplementary Table S2), which are the negatively charged RNA atoms that are often compensated for by the positive charge of Mg^2+^. The only other types of nucleotide oxygen atoms with inner-sphere *F*_atom_ ≥ 1 are two types of keto-oxygens (U-O4, G-O6) from nucleobase (O_b_). The *F*_atom_ value of the most frequently observed O_b_ ligand atom (U-O4) is just two times smaller than the value for O_ph_ atoms, even though U-O4 lacks the ability to significantly compensate for the positive charge of Mg^2+^. Not all O_b_ atoms exhibited similar normalized frequency to serve as ligands for Mg^2+^ binding. The *F*_atom_ values for O_b_ atoms adjacent to the ribose bond (C-O2 and U-O2) were up to 290 times lower than that for O_b_ atoms located opposite to the sugar edge (G-O6 and U-O4) (Figure 3). The reason is that in most binding site configurations, the ribose blocks the adjacent oxygen atom (C-O2 or U-O2) from binding Mg^2+^ by steric clashes between Mg^2+^ inner-sphere waters and the ribose. Unlike O_ph_ and some O_b_ atoms, ribose oxygen atoms (O_r_) are rarely found in Mg^2+^ inner sphere.

Besides oxygen atoms, nitrogen atoms from nucleobases (N_b_) are the only other RNA atoms to serve as inner-sphere ligands, but generally they were observed in the inner sphere less frequently (Figure 3). Out of all six types of N_b_ atoms with a lone electron pair in the plane of the aromatic ring (–N=), only two (G-N7 and A-N7) have an inner-sphere *F*_atom_ value higher than one. All other –N= atoms were almost never observed to coordinate Mg^2+^ in the inner sphere.

Though outer-sphere hydrogen bonds are chemically different from the inner-sphere coordination bonds, the most frequent inner-sphere atoms (O_ph_, U-O4, G-O6, G-N7 and A-N7) are also among the most frequent outer-sphere atoms of Mg^2+^ (Figure 3). However, the order of the relative frequencies differs and O_ph_ atoms are no longer the most frequently observed. The most frequent outer-sphere atoms are G-O6 and G-N7, followed by O_ph_, U-O4 and A-N7. Similar to the inner sphere, the frequencies of outer-sphere interactions for O_b_ atoms located opposite to the sugar edge (G-O6 and U-O4) are much greater than those for O_b_ atoms adjacent to the ribose bond (C-O2 and U-O2). In spite of the similar high frequencies of certain atoms to coordinate Mg^2+^ both through the outer and inner sphere, several types of atoms rarely found in the inner sphere were found to form outer-sphere hydrogen bonds quite frequently (Figure 3); most notably the O3′ and O5′ atoms from ribose and A-N6 and C-N4 from the exocyclic amino groups of nucleobases (–NH_2_). A more extensive discussion of the differences in atomic Mg^2+^ coordination frequencies is presented in Supplementary Text 2.

Guanine is the most frequently observed nucleobase to coordinate Mg^2+^ in the inner sphere due to the combined effect of two atoms that have high *F*_atom_ values (G-O6 and G-N7). The inner-sphere *F*_atom_ value for U-O4 is higher than that for A-N7, which makes uracil the second most frequently observed nucleobase. As a net result, the frequency for nucleobases to coordinate Mg^2+^ in the inner sphere is (in descending order) G > U > A > C. In the outer sphere, the guanine moiety is also the most frequently observed nucleobase, followed by adenine and uracil, exhibiting a slightly different trend (G > A > U > C) than is observed for the inner sphere.

### Inner- and outer-sphere composition of Mg^2+^ sites

**Figure 4.**
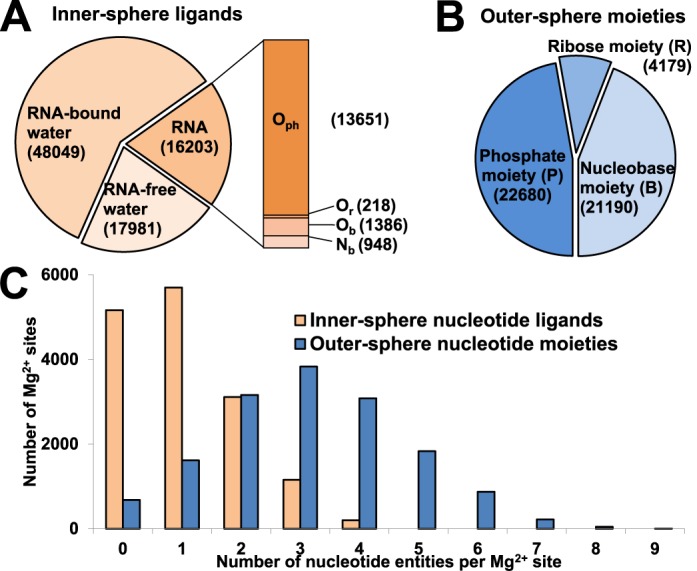
Distribution of Mg^2+^ ligands in the benchmark dataset. (**A**) The occurrence (number of interactions) of RNA atoms, RNA-bound water and RNA-free water in the Mg^2+^ inner sphere. RNA-bound water is defined as an inner-sphere water molecule that forms hydrogen bond(s) with RNA atom(s). RNA-free water is defined as an inner-sphere water molecule found within RNA-bound Mg^2+^ sites but not forming direct hydrogen bonds with RNA atoms. Mg^2+^ sites with only water molecules in both the inner and outer sphere were not considered as RNA-bound Mg^2+^ sites and therefore are not included in the benchmark dataset. (**B**) Occurrence of phosphate, ribose and nucleobase moieties in the Mg^2+^ outer sphere. (**C**) Per-site distribution of inner-sphere nucleotide atoms (orange) and outer-sphere nucleotide moieties (blue) in Mg^2+^ sites.

Nucleotide ligands represent one-fifth of all inner-sphere Mg^2+^ interactions in the benchmark dataset (Figure 4A). The majority of inner-sphere ligands are water molecules. Water molecules in the inner sphere without direct hydrogen bonds to RNA (17981 instances) are less common than those bound to RNA (48049 instances). In the outer sphere, nucleobase moieties are almost as abundant as phosphate moiety. The ribose moiety is much less common in the outer sphere (Figure 4B).

The Mg^2+^-binding environments were surveyed for the number of inner-sphere ligands contributed by nucleotides and the number of outer-sphere nucleotide moieties per individual site (Figure 4C). Thirty-one percent of the sites do not have any inner-sphere nucleotide ligands. The number of sites decreases gradually as the number of nucleotide ligands per site increases. The maximum number of nucleotides contributing inner-sphere ligands to a Mg^2+^ site is 4, but such cases are very rare.

The distribution of the number of outer-sphere nucleotide moieties per site peaks at 3, though the outer spheres of Mg^2+^ sites frequently accommodate up to six moieties. A few ‘overcrowded cases’ with 7–9 moieties have been observed, though these are very rare (Figure 4C). The vast majority of the sites in the benchmark dataset feature at least one or more outer-sphere nucleotide moieties.

### Overview of Mg^2+^ site classification

All Mg^2+^ sites in the benchmark dataset were divided according to their binding environment into four mutually exclusive classes in the following order (Table 1): (i) sites with additional metal ion(s) within 4 Å from Mg^2+^; (ii) sites with non-RNA, non-water atoms in the inner sphere; (iii) sites with only RNA atoms as non-water ligands in the inner sphere (RNA-inner) and (iv) sites with only water molecules in the inner sphere and at least one RNA moiety in the outer sphere (RNA-outer).

**Table 1.** Numbers of Mg^2+^ sites included in the benchmark dataset for the four site classes described in the text

Dataset	Total	Metal atom within 4 Å of Mg^2+^	Protein or small molecule in Mg^2+^ inner sphere	RNA-inner	RNA-outer
All data	15334	412 [2.7%]	268 [1.7%]	9628 [63%]	5026 [33%]
Ribosome	14682	373 [2.5%]	195 [1.3%]	9382 [64%]	4732 [32%]
Non-ribosome	652	39 [6.0%]	73 [11%]	246 [38%]	294 [45%]

The percentage of sites in each site class is shown in brackets.

**Figure 5.**
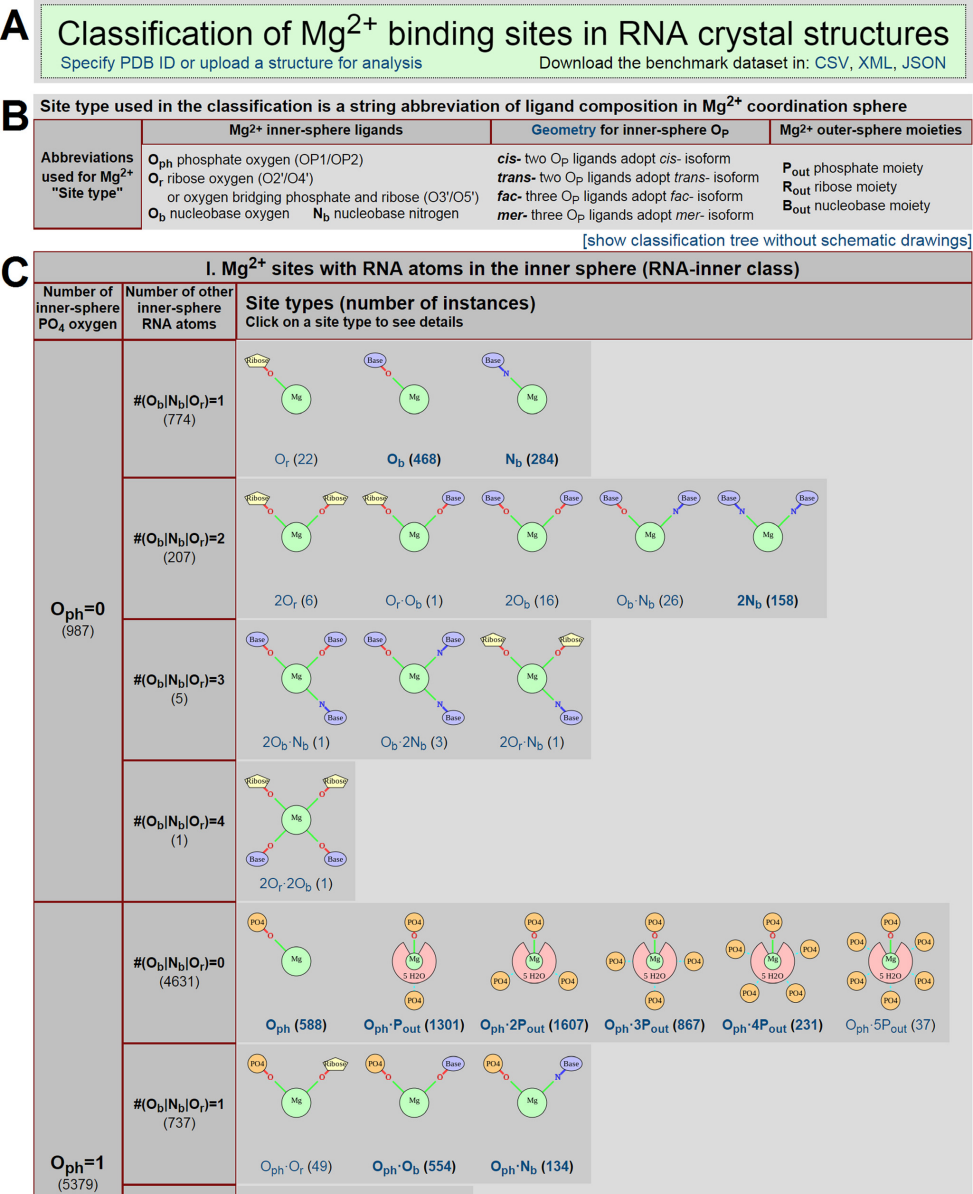
Screenshot of the server showing the structural classification of Mg^2+^ sites in the benchmark dataset. (**A**) The title box contains a link for specifying a particular PDB ID or uploading a PDB file for the quality analysis and classification of Mg^2+^ sites, as well as links to download the benchmark dataset in various formats. (**B**) The summary of terminology used in defining Mg^2+^ binding sites. (**C**) A fraction of the main classification table reveals a tree-like classification of Mg^2+^ sites at three different levels represented by columns. The schematic drawings used to visualize each site type can be switched off using a link above the top-right corner of the table to reveal a brief overview of the classification tree. Each schematic drawing contains an embedded link to all the sites in the benchmark dataset that are classified into that site type.

Classes (i) and (ii) comprise only a small fraction of sites in the benchmark dataset (2.7% and 1.7%, respectively; Table 1). The RNA-inner and RNA-outer classes represent the majority (96%) of sites in the benchmark dataset (Table 1). Therefore only these two classes were further classified into 136 types based on the structural arrangements of their binding environments (Supplementary Tables S3/S4) in this manuscript. The detailed classification of the RNA-inner and RNA-outer classes is accessible at http://www.csgid.org/metalnas/ (Figure 5).

### RNA-inner sites classification

The RNA-inner class is the most populous class, representing 63% of the benchmark dataset (Table 1). Of the four types of inner-sphere atoms (O_ph_, O_r_, O_b_ and N_b_), O_ph_ is most abundant (Figure 3) and is likely to contribute most to the energy of Mg^2+^ binding via its distinct ability to compensate for the ion's charge. Hence, the number of inner-sphere O_ph_ atoms was the first criterion chosen to classify RNA-inner site types (Supplementary Table S3), while the total number of other inner-sphere atoms (O_r_|O_b_|N_b_) was chosen to be the second criterion. The maximum number of RNA atoms serving as inner-sphere ligands is 4 per site (Figure 4C), so combining the first and second criteria resulted in 14 possible subclasses (Supplementary Table S3). These 14 combinations were further divided into 41 types of Mg^2+^ binding sites based on specific types of O_r_|O_b_|N_b_ atoms and the geometrical isomerism of O_ph_ atoms (*cis-/trans-/mer-/fac-*) if more than one O_ph_ is present. For the most populated branch (#O_ph_ = 1, #O_r_|O_b_|N_b_ = 0), the number of outer-sphere phosphates was also taken as an additional criterion to subdivide sites.

The general trend seen in the inner-sphere *F*_atom_ values for nucleobase and ribose atoms (O_b_>N_b_>O_r_, Figure 3B) is also observed in the population of different site types within the same subclass (i.e. with the same number of inner-sphere O_ph_ and O_r_|O_b_|N_b_ atoms). The only exception to this trend is the 2N_b_ type in the #O_ph_ = 0, #(O_r_|O_b_|N_b_) = 2 subclass, which contains a disproportionately high population (158 sites). All other types with more than one O_r_|O_b_|N_b_ inner-sphere ligand, regardless of subclass, contain 28 sites or less. Both *fac-* and *mer-* conformations have similar abundances for sites with #O_ph_ = 3, while the *cis-* conformation is more frequently observed than the *trans-* conformation for all #O_ph_ = 2 subclasses. Eighty-six percent of the sites in the subclass #O_ph_ = 2, #(O_r_|O_b_|N_b_) = 0 are in the *cis*- conformation. The *cis*-2O_ph_ conformation is even more predominant (283 out of 286 sites) in the sites in which non-O_ph_ inner-sphere ligands are also present (subclasses #O_ph_ = 2, #(O_r_|O_b_|N_b_) = 1,2).

### RNA-outer site classification

The RNA-outer class represents a significant fraction (33%) of all sites in the benchmark dataset (Table 1). The outer-sphere composition was used to subdivide the class due to the absence of inner-sphere RNA atoms (Supplementary Table S4). Instead of employing individual *interactions* as was done for the inner sphere, individual outer-sphere *moieties* were used to subdivide RNA-outer sites. We believe that the number of moieties represents the uniqueness of a given structural arrangement of RNA, while the actual number of interactions (hydrogen bonds) of the moiety with inner-sphere water molecules just slightly contributes to the energy of binding and may easily vary between very similar sites. Similar to RNA-inner site classification, the number of outer-sphere phosphate moieties #P_out_ was chosen as the first criterion and the total number of ribose and nucleobase moieties #(R_out_/B_out_) was used as the second criterion to subdivide the RNA-outer class, resulting in 39 subclasses. The specific combinations of ribose (#R_out_) and nucleobase (#B_out_) moieties in the outer sphere produced a list of 95 types of RNA-outer sites (Supplementary Table S4).

The most populous RNA-outer site type is 2B_out_ with only two nucleobase moieties in its outer sphere (555 sites). This site type is often found in RNA helices, as base stacking results in very close positions of two consecutive bases coordinating one Mg^2+^. Site types containing only phosphate moieties are less populous yet still abundant, represented by P_out_ (94 sites), 2P_out_ (191 sites), 3P_out_ (207 sites) and 4P_out_ (174 sites). The rarity of the ribose moiety in the outer sphere (Figure 4B) was reflected in the low populations of R_out_-containing types, not exceeding 60 instances per type.

### Detection of previously reported Mg^2+^-binding motifs

In the current study, we sought to identify and analyze ‘validated motifs,’ which we considered to be a specific structural arrangement provided by RNA for Mg^2+^ binding, which is found in structures of multiple RNA molecules. Most Mg^2+^-binding motifs reported previously, including some of those annotated in MeRNA (13), were based on the analysis of a single RNA structure (2,7–12). Therefore, it remains to be verified if these reported architectures could be found in other structures or whether they were a unique feature in a given structure.

The systematic site classification presented in this paper was used to describe six validated Mg^2+^-binding motifs previously reported in the literature (Table 2, Supplementary Figure S5). A site type was defined for each literature-derived motif, and a few additional criteria were applied to specify the motif precisely within the site types. For example, the two O_P_ ligands that define the ‘magnesium clamp’ (7,30) must be from different chains or from nucleotides separated by more than seven residues from one another (Table 2). Those additional criteria were implemented in the form of database queries which retrieved all the sites from the benchmark dataset that fulfill the requirements. The validity of our motif definitions was further verified by the capability of these customized queries to locate the Mg^2+^ binding sites reported in the original literature. Certain previously reported motifs involving specific RNA structures with a given type of Mg^2+^ binding site were rarely observed and therefore were not considered a ‘validated motif’ herein, such as G–A pair (5,9), sheared G–A pairs, A-rich bulge and the three helix junction (2).

**Table 2.** Three RNA-inner and three RNA-outer validated Mg^2+^-binding motifs previously reported in the literature (I–VI)

ID	Motif name	Reference	Systematic classification		Detailed features of the motif	Number of sites
			Class	Type		
I	Magnesium Clamp	Ennifar *et al*. (1999) (7) Petrov *et al*. (2011) (30)	RNA-inner	*cis*-2O_ph_*trans*-2O_ph_	The two O_ph_ atoms are from distant phosphates	823 (*cis*)207 (*trans*)
II	10-member ring	Hsiao *et al*. (2009) (12)	RNA-inner	*cis*-2O_ph_	The two O_ph_ atoms are from sequentially consecutive phosphates	802
				*fac*-3O_ph_ *mer*-3O_ph_	The three O_ph_ atoms are from sequentially consecutive phosphates	63 (*fac*) 39 (*mer*)
				*fac*-3O_ph_*mer*-3O_ph_	10-member ring with additional O_ph_ atom coming from phosphate which is separated by one residue from either 10-member ring phosphates	10 (*fac*)74 (*mer*)
				4O_ph_	One Mg^2+^ coordinated by two unrelated 10-member rings	37
III	G-phosphate	Klein *et al*. (2004) (10)	RNA-inner	O_ph_•O_b_	O_b_ atom is from guanine	250
IV	G•G metal-binding site	Correll *et al*. (1997) (8)	RNA-outer	2P_out_•2B_out_	Mg^2+^ is bound through the outer sphere by two sequentially consecutive guanines and by two phosphate moieties from residues n and n-1 where n is residue number of the lower guanine in the sequence.	95
		3OFA-A1569		2B_out_	Mg^2+^ is bound through the outer sphere by two sequentially consecutive guanine moieties	374
		3V23-A3619		P_out_•2B_out_	Mg^2+^ is bound through the outer sphere by two consecutive guanines and a distant phosphate	57
V	Triple G Motif	Tinoco & Keift (1997) (2)	RNA-outer	3B_out_	Mg^2+^ is bound through the outer sphere by three sequentially consecutive guanine bases	34
VI	Metal ion zipper	Correll *et al*. (1997) (8)	RNA-outer	2P_out_	Mg^2+^ binds two distant phosphates through the outer sphere	97

The site type(s) and additional features used to define each motif are tabulated. The term ‘distant’ phosphates/residues is used to specify two phosphates or residues coming from different RNA chains or separated by more than seven residues in the same chain. The terms ‘upstream’/‘downstream’ residues are used to specify a residue lower/higher in the sequence of the same chain. Representative examples of each motif are depicted in Supplementary Figure S5.

All six validated motifs are reasonably abundant in the benchmark set, with the number of instances ranging between 34 for the Triple-G motif (2) and 1030 for the magnesium clamp (7,30). The 10-member-ring motif (12), with two inner-sphere O_P_ atoms from consecutive nucleotides forming the core of the motif, is found in four different variants. The G-phosphate motif (10) and metal ion zipper (8) were found only in rRNA.

### Identification of novel Mg^2+^-binding motifs

The classification clusters Mg^2+^ sites with similar RNA interaction patterns, which allows a search for novel validated motifs. Selected sites from some of the more populated site types were investigated visually for the presence of a recurring specific pattern in a variety of structures. For each found pattern a respective database query was created to screen the benchmark dataset and retrieve all sites with that specific pattern. This motif-discovery approach resulted in the detection of seven validated motifs, five in the RNA-inner class and two in the RNA-outer class (Table 3).

**Table 3.** Seven newly discovered and validated Mg^2+^-binding motifs

ID	Motif name	Representative site	Systematic classification		Detailed features of the motif	Number of sites
			Class	Type		
A	Y-clamp	2Z75-B301	RNA-inner	*mer*-3O_ph_	10-member ring and one distant O_ph_ atom in *mer-* conformation	279
B	U-phosphate	2YIE-Z1116	RNA-inner	O_ph_•O_b_	O_b_ atom is from uracil	199
C	12-member ring	2AVY- A1566	RNA-outer	2P_out_	Mg^2+^ is bound through the outer sphere by two sequentially consecutive phosphates forming a ring with 12 non-hydrogen atoms	82
D	Purine-N7 seat	2QBA-B3321	RNA-inner	2N_b_	Mg^2+^ is bound by two guanine or adenine N7 atoms in inner sphere and capped by 3 or 4 downstream phosphates through the outer sphere	141
E	G-N7 macro-chelate I	3V29-A3359	RNA-inner	N_b_•P_out_	Mg^2+^ is bound by guanine N7 atom in inner sphere and phosphate of the same residue in the outer sphere	60
F	G-N7 macro-chelate II	2AW7-A1569	RNA-outer	P_out_•B_out_	Mg^2+^ is bound through the outer sphere by guanine N7 atom and phosphate of the same residue	18
G	10-member ring with purine-N7	3V23-A3540	RNA-inner	2O_ph_•N_b_	10-member ring with N7 atom of purine base which is separated by one residue from either of the 10-member ring phosphates	45

**Figure 6.**
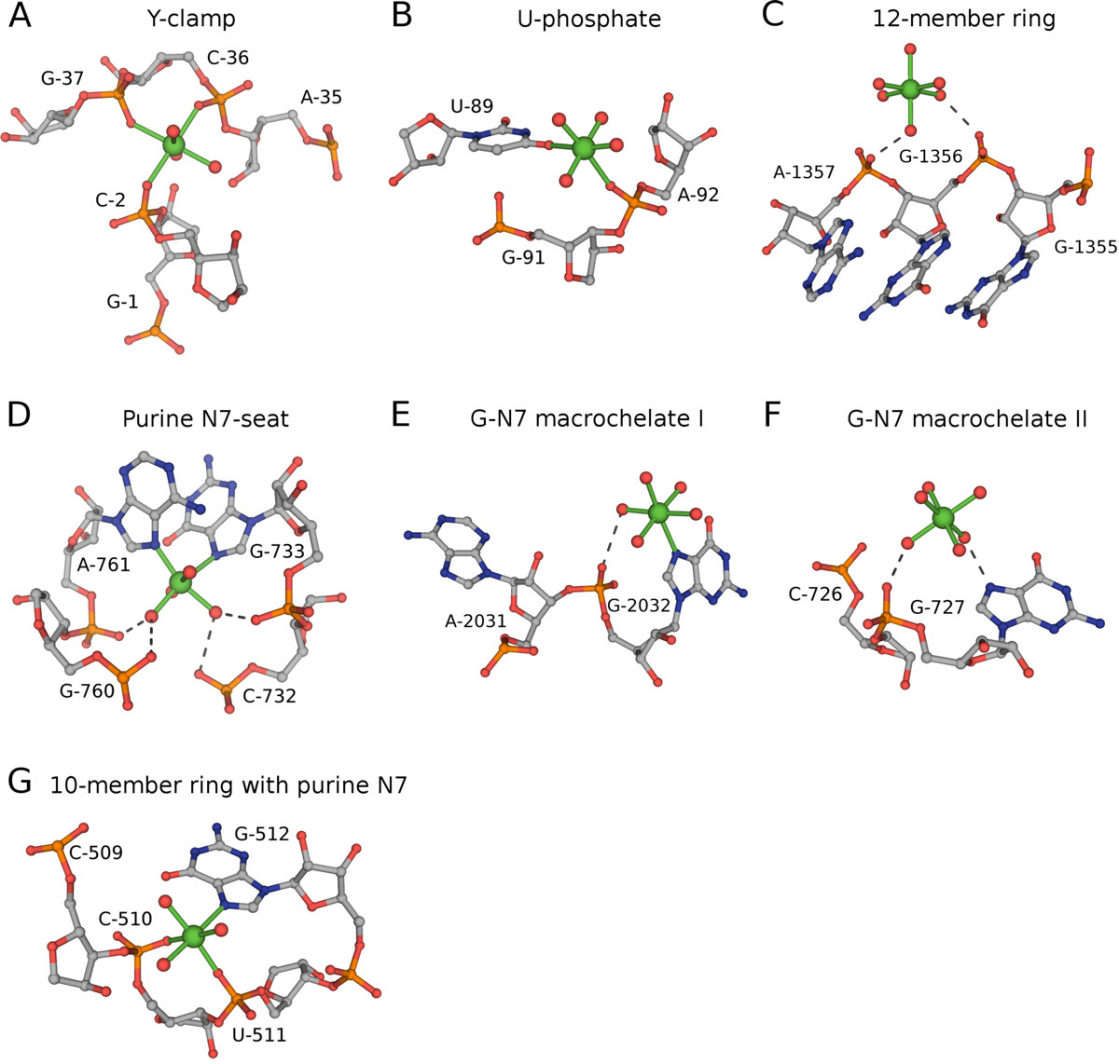
Newly discovered and validated Mg^2+^-binding motifs. Magnesium ions and inner-sphere interactions are shown in green. Outer-sphere hydrogen bonds are shown as gray dashed lines. The nucleotides forming the motifs are labeled. The figure was prepared using PyMol. The representative sites defined by PDB codes, Mg^2+^ residue number, structure resolution and validation Q-values (*Q_v_* / *Q_s_* / *Q_e_*) are as follows: (**A**) 2Z75-B301, 1.7 Å (0.8 / 0.9 / 0.9); (**B**) 2YIE-Z1116, 2.94 Å (0.7 / 0.8 / 1.0); (**C**) 2AVY- A1566, 3.46 Å (1.0 / 1.0 / 1.0); (**D**) 2QBA-B3321, 3.54 Å (1.0 / 0.9 / 0.9); (**E**) 3V29-A3359, 3.1 Å (0.8 / 0.9 / 0.8); (**F**) 2AW7-A1569, 3.46 Å (1.0 / 1.0 / 0.5); (**G**) 3V23-A3540, 3 Å (0.6 / 0.9 / 0.9).

The PDB accession codes with the residue number for the magnesium ion are shown for representative sites of each motif (as depicted in Figure 6). Site type and additional features used to define each motif are tabulated.

The ‘Y-clamp’ motif (Figure 6A), which is observed in both ribosomal subunits, riboswitch and ribozyme, stabilizes RNA structures by anchoring two different strands or two distant parts of the same strand together in a similar manner as the magnesium clamp (7). The letter ‘Y’ in the name of the motif was chosen to resemble the three-way configuration of inner-sphere O_P_ atoms, while ‘clamp’ refers to the bridging capability of phosphates by Mg^2+^. This unique feature for maintaining an RNA fold, which resembles the disulfide bridge linkage in proteins, is very common in RNA structures: 814 magnesium clamps and 238 Y-clamps were found. The ‘U-phosphate’ motif (Figure 6B) resembles the previously reported G-phosphate motif (10), save that uracil is substituted for guanine. Similar to the G-phosphate motif, this motif was found mostly in ribosome. The ‘12-member ring’ RNA-outer motif involves two outer-sphere phosphate moieties from consecutive residues in the RNA backbone (Figure 6C). This motif is similar to the ‘10-member ring’ RNA-inner motif, save that it has outer-sphere instead of inner-sphere interactions. Even though not further explored, multiple variations of the ‘12-member ring’ motifs are expected similarly to the multiple variations reported for 10-member ring motifs (12).

Four more validated motifs were found exclusively in ribosome. The ‘purine N7-seat’ motif contains a very characteristic coordination pattern formed by inner-sphere nucleobases (2N_b_) and outer-sphere phosphate moieties (Figure 6D). The existence of this motif results in a disproportionately high population of the 2N_b_ type as observed in the benchmark dataset (Supplementary Table S3). The N7 atoms serving as ligands in motif are usually found in two guanine bases, but sometimes an adenine-guanine pair was found. Two single-nucleotide and guanine-specific ‘macrochelate’ motifs were identified with G-N7 serving as a ligand in either inner sphere or outer sphere, and ‘macrochelated’ with an outer-sphere phosphate moiety (Figure 6E and F). Similar ‘macrochelate’ patterns have been previously reported for structures of mononucleotides (11,31). A specific motif named ‘10-member ring with Purine-N7’ was found to have a 10-member ring together with an additional ligand formed by the N7 atom of a purine base, which is separated by one residue from either of the 10-member ring phosphates (Figure 6G).

## DISCUSSION

### Validation strategy

Due to the poor quality of many RNA crystal structures deposited in the PDB, validation is a critical step for any structural data mining study to have biological relevance. Unfortunately, the common practice of using resolution as the main selection criteria to define a ‘good’ dataset is infeasible for our analysis for two reasons. First, the high flexibility of RNA and large unit cell dimensions of RNA crystals means that many structures of RNA are of relatively poor resolution—the majority of magnesium ions in the PDB are found in structures of 2.5 Å resolution or worse (Figure 1A). Second, even in high-resolution structures, a substantial fraction of Mg^2+^ are still significantly under-coordinated (Figure 1A) and therefore questionable. To account for the significant errors in atomic positions present at low resolution (32), a unique two-step search of inner-sphere ligands was employed to conditionally allow a generous Mg^2+^-ligand distance deviation of up to 1.0 Å for selected favorable ligands, yet limit the inclusion of interactions unlikely to be specific. Tailored for a whole range of resolutions, the inner-sphere definition used herein recognizes ligands more specifically than do the algorithms for other metal databases which use a simple distance cutoff (13,14,33–35).

Since the scope of this study was Mg^2+^ sites in RNA structures, which informed the selection of the benchmark dataset, only RNA-bound (through either inner or outer sphere) sites were accepted in order to exclude those Mg^2+^ sites which are bound only to protein in RNA–protein complexes or do not have any clear connection to RNA. The Mg^2+^ validation procedure was largely based on the similarity of each site to the rigid octahedral arrangement of inner-sphere ligands expected for Mg^2+^ and the characteristically short Mg^2+^-ligand distances. Harnessing those intrinsic properties for Mg^2+^ identification required that all sites have a relatively complete coordination sphere; therefore only sites with CN = 4–6 were accepted. The three validation parameters used to select sites for the benchmark dataset evaluated Mg^2+^-ligand distances (*Q_v_*), the symmetry of the inner-sphere ligands arrangement (*Q_s_*) and the agreement of Mg^2+^ B-factors with the surrounding atoms (*Q_e_*). Using the combination of multiple parameters as filtering criteria effectively removed the majority of poorly modeled Mg^2+^ sites. However, a few Mg^2+^ sites with chemically infeasible interactions were still not caught by the criteria, for example, those with amino nitrogen atoms in the inner sphere. To exclude those cases, we introduced an additional criterion for sites with nitrogen atoms from nucleobases in the inner sphere: namely, accepting only those sites which have an endocyclic nitrogen with a lone electron pair in the plane of the aromatic ring (–N=) since only this type of nitrogen from nucleobase can feasibly coordinate Mg^2+^ (Supplementary Text 2).

Some potential Mg^2+^ binding sites in RNA are absent from the benchmark dataset because the electron density for Mg^2+^ was not observed, or density was present but was incorrectly modeled as another metal ion or as a water molecule. Other sites might have had a true magnesium ion, but an incompletely modeled inner sphere resulted in some of the validation parameters being below the benchmark dataset thresholds. In the latter case, the inner sphere can often be completed by placing additional water molecules during crystallographic re-refinement of problematic structures. In this way, the benchmark dataset can be extended by revealing additional ‘trustworthy’ Mg^2+^ sites, but would require manual inspection of each site and is beyond the scope of the current study. Nevertheless, our algorithms enabled extraction of many existing trustworthy Mg^2+^ sites which can be used for further statistical and classification studies. For example, the benchmark dataset is particularly valuable to be used as a training dataset for tools to predict the positions of Mg^2+^ ions in both experimental and theoretical models of RNA structures and/or increase the accuracy of Mg^2+^ prediction. Thus far, we have implemented the benchmark dataset developed in this work as one of the alternative reference datasets in MetalionRNA predictor at http://metalionrna.genesilico.pl/ (36).

### Crystallographic model-building artifacts

The reliability of a Mg^2+^ binding site is highly dependent on modeling strategy and whether restraints were properly used during crystallographic refinement. The distance distribution of Mg^2+^-water distances reveals the presence of two large sharp peaks which originate from strongly restrained sites at correct (2.08 Å) or incorrect (2.18 Å) Mg^2+^-water distances used in some refinement programs by default (Supplementary Figure S4A). (We use ‘correct’ or ‘incorrect’ in the sense that the values do or do not agree with the mean distances observed in atomic resolution small molecular crystal structures.) Around 200 strongly restrained Mg^2+^ sites at another incorrect (1.83 Å) Mg^2+^-water distance were also identified (Supplementary Figure S4A) in a 40S ribosomal subunit (PDB codes 2XZM, 2XZN) (29). On the contrary, the absence of prominent peaks in the distributions of Mg^2+^−O (non-water) and Mg^2+^-N distances, even in sites that satisfy the validation criteria, suggests that those interactions are loosely restrained during crystallographic refinement (Supplementary Figure S4B and C).

The proper use of Mg^2+^-ligand distance restraints is essential for correct modeling of a Mg^2+^ inner sphere, especially of low-resolution structures. However, improperly strict or weak restraints may result in misinterpretation of experimental data, and thus in turn impair subsequent research. Given the prevalence of poorly modeled Mg^2+^ sites in RNA structures and improper use of crystallographic restraints (either too strict or too loose), we propose that the crystallographic refinement software should include easy-to-use restraints to enforce correct CN and geometrical arrangement of ligands around metal ions.

### Benchmark dataset redundancy

The main objective in the construction of the benchmark dataset was ensuring the reliability of the Mg^2+^ binding sites. Redundant structures (i.e. having even 100% identical nucleotide sequences) were not excluded. It is beneficial to include sites that satisfy validation criteria from all structures in the analysis because different structures of the same macromolecule may carry different sets of reliable Mg^2+^ sites due to differences in diffraction data quality, refinement strategies, crystallization conditions, bound ligands and/or macromolecule conformation. Moreover, it is essential to preserve as much variety of the different coordination patterns that may be present for otherwise equivalent Mg^2+^ sites observed in different PDB deposits of homologous RNA molecules.

Even though a certain level of redundancy was observed in the benchmark dataset, all further analyses were carefully designed to control for the presence of redundancy. The statistics used for estimation of the preferences of atoms and nucleotides to bind Mg^2+^ are minimally affected by dataset redundancy, because the frequencies of individual interactions are normalized by the frequency of atoms or nucleotides in the dataset. Dataset redundancy is beneficial for the classification because it ensures that the architecture of each reasonable Mg^2+^ site was accounted for. The numbers of instances of each validated Mg^2+^ motif do indeed contain redundant sites, but each motif was manually confirmed to represent a variety of sites in non-redundant structures.

### Magnesium ion binding preferences

The *F*_atom_ values produced by statistical analysis of the benchmark dataset indicate the frequency at which certain types of RNA atoms served as ligands for Mg^2+^ in the inner or outer coordination sphere, and are normalized so that values for different atoms can be directly compared to one another. These values are consistent with the steric accessibility and chemical properties of each atom type (Supplementary Text 2), and we propose that these frequencies are reasonable estimates of the ‘propensity’ or ‘preference’ of these RNA atom types to coordinate Mg^2+^. However, we cannot formally rule out the possibility of sampling bias (i.e. the binding sites in the benchmark set may not be wholly representative of Mg^2+^ binding in RNA universally). For example, the analyzed set was necessarily limited to RNA molecules which form diffraction-quality crystals and, as mentioned above, may exclude sites that were incompletely or incorrectly modeled. The values of *F*_atom_ (and the preferences they imply) can be used as prior knowledge for predicting ‘probable’ versus ‘improbable’ Mg^2+^ sites in both computational modeling and crystallographic refinement.

Even if the main role of Mg^2+^ is believed to be neutralization of the negative charge of phosphate moieties in RNA (2), nucleobase moieties were shown to be relatively abundant in the inner sphere and are almost as abundant as phosphate moieties in Mg^2+^ outer spheres (Figure 4). Therefore the coordination of Mg^2+^ by nucleobases should be considered as a significant factor in the stabilization of RNA structure. Our data suggests (Figure 3B) that guanosine nucleotide has a more pronounced effect on RNA structure stabilization than any other nucleotide due to its predominance in Mg^2+^ binding, which is supplemented by its ability to form a greater number of hydrogen bonds in base pairing.

### Mg^2+^ site classification and motif description

The classification system is based on a simple dendrogram-like hierarchy of Mg^2+^ interactions, with the number of ligands and their chemical differences serving as the main criteria to define branches. This classification system was designed to be readily understandable and easy to automate, to make it an efficient tool for investigation of the diversity of Mg^2+^ binding sites. The number of site types resulting from the classification strategy is within a reasonable range for practical usage; i.e. too many site types would render the system difficult to use, whereas too few types would be not enough to distinguish motifs and specific sites. The 136 site types in the RNA-inner and RNA-outer classes observed in the benchmark dataset used herein is not an exhaustive list; as additional RNA structures are determined, new site types are likely to be discovered. The hierarchical classification system offers different levels of abstraction depending on the particular application. For example, the use of just the number of inner-sphere phosphates as a determinant of site family yields only five families in the RNA-inner class. The naming convention of site types explicitly spells out the types and number of all ligands (or outer-sphere moieties in the case of the RNA-outer class) present in the site type. The name of each site type is unique and contains enough information to infer the subclasses at broader levels of classification.

The practical use of the classification system has been shown by the precise definition of previously reported Mg^2+^-binding motifs, which (along with just a few additional criteria) is sufficient to identify new instances of these motifs (Table 2) and by the discovery of entirely novel motifs (Table 3). Both the previously reported and the newly found Mg^2+^-binding motifs highlighted the ubiquitous presence of outer-sphere interactions, which play vital roles in inducing and maintaining the proper folding and formation of the Mg^2+^-binding pocket. Three out of the six literature-reported motifs are defined by outer-sphere interactions exclusively (Table 2). As for the seven newly discovered motifs, two of them involve only outer-sphere interactions, while two others involve both characteristic inner- and outer- sphere interactions (Table 3). Therefore, comprehensive handling of outer-sphere interactions is of indispensable importance for the description of Mg^2+^-binding motifs.

### Future applications of Mg^2+^ site classification

Although a lot of Mg^2+^-binding motifs are expected to play mostly a universal structural role by charge compensation and fold stabilization, the presence of some Mg^2+^ structural motifs may also indicate functional implications. A preliminary study has been carried out toward this direction, by correlating RNA functional families with site type as defined in our classification system. We noticed that inner-sphere interactions are more abundant in the large ribosomal subunit than in the small ribosomal subunit. We also noticed that base stacking is more frequent in the outer sphere of Mg^2+^ sites in ribozyme. However, further study will be necessary to find more detailed correlations.

We believe our classification system has potential to detect unique structural sites responsible for specific functional roles. Mg^2+^ binding sites with unique and complicated structural arrangements, especially those that are rarely observed, may be good candidates for investigation. Our tables of the populations of site types (Supplementary Tables S3/S4) and the server that describes them may be used by researchers to determine the extent of uniqueness of a particular coordinating pattern, allowing them to highlight candidate Mg^2+^ sites for specific consideration.

With the availability of more data in the future, the method used may provide evidence of new validated Mg^2+^ motifs, i.e. some specific coordinating patterns will prove to be sufficiently populated. Moreover, increasing the number of sites could allow a detailed classification of more complicated sites with additional metal or non-RNA ligand in the Mg^2+^ coordination sphere. The growth of structural data will also permit the systematic investigation of other metals less commonly observed in RNA.

### Online access

The classification of Mg^2+^ sites in the benchmark dataset can be accessed via URL http://www.csgid.org/metalnas/. The main page of the server lists all site types with schematic drawings and the number of sites found for each site type. Detailed information for each site type may be accessed, which includes an image of a representative site and a list of all PDB entries containing a site of that type with chain and residue ids. Each Mg^2+^ site may be visualized in *Jmol* (37). Users can specify a particular PDB ID or upload a RNA structure in the PDB format for analysis. A simple REST API is provided for users to download the whole benchmark dataset, as well as results of each particular search or analysis, in various formats (Figure 5A).

## SUPPLEMENTARY DATA

Supplementary Data are available at NAR online.

SUPPLEMENTARY DATA

## References

[1] Draper D.E. (2008). RNA folding: Thermodynamic and molecular descriptions of the roles of ions. Biophys. J..

[2] Tinoco I. Jr, Kieft J.S. (1997). The ion core in RNA folding. Nat. Struct. Biol..

[3] Jenner L., Demeshkina N., Yusupova G., Yusupov M. (2010). Structural rearrangements of the ribosome at the tRNA proofreading step. Nat. Struct. Mol. Biol..

[4] Brannvall M., Kirsebom L.A. (2001). Metal ion cooperativity in ribozyme cleavage of RNA. Proc. Natl Acad. Sci. U.S.A..

[5] Scott W.G., Finch J.T., Klug A. (1995). The crystal structure of an all-RNA hammerhead ribozyme: a proposed mechanism for RNA catalytic cleavage. Cell.

[6] Bowman J.C., Lenz T.K., Hud N.V., Williams L.D. (2012). Cations in charge: magnesium ions in RNA folding and catalysis. Curr. Opin. Struct. Biol..

[7] Ennifar E., Yusupov M., Walter P., Marquet R., Ehresmann B., Ehresmann C., Dumas P. (1999). The crystal structure of the dimerization initiation site of genomic HIV-1 RNA reveals an extended duplex with two adenine bulges. Structure.

[8] Correll C.C., Freeborn B., Moore P.B., Steitz T.A. (1997). Metals, motifs, and recognition in the crystal structure of a 5S rRNA domain. Cell.

[9] Baeyens K.J., De Bondt H.L., Pardi A., Holbrook S.R. (1996). A curved RNA helix incorporating an internal loop with G.A and A.A non-watson-crick base pairing. Proc. Natl Acad. Sci. U.S.A..

[10] Klein D.J., Moore P.B., Steitz T.A. (2004). The contribution of metal ions to the structural stability of the large ribosomal subunit. RNA.

[11] Lippert B. (2000). Multiplicity of metal ion binding patterns to nucleobases. Coordin. Chem. Rev..

[12] Hsiao C., Tannenbaum E., VanDeusen H., Hershkovitz E., Perng G., Tannenbaum A.R., Williams L.D., Hud NV (2009). Complexes of nucleic acids with group I and II cations. Nucleic Acid-Metal Ion Interactions.

[13] Stefan L.R., Zhang R., Levitan A.G., Hendrix D.K., Brenner S.E., Holbrook S.R. (2006). MeRNA: a database of metal ion binding sites in RNA structures. Nucleic Acids Res..

[14] Schnabl J., Suter P., Sigel R.K. (2012). MINAS–a database of metal ions in nucleic AcidS. Nucleic Acids Res..

[15] Berman H.M., Westbrook J., Feng Z., Gilliland G., Bhat T.N., Weissig H., Shindyalov I.N., Bourne P.E. (2000). The protein data bank. Nucleic Acids Res..

[16] Cooper D.R., Porebski P.J., Chruszcz M., Minor W. (2011). X-ray crystallography: assessment and validation of protein-small molecule complexes for drug discovery. Expert Opin. Drug Discov..

[17] Pozharski E., Weichenberger C.X., Rupp B. (2013). Techniques, tools and best practices for ligand electron-density analysis and results from their application to deposited crystal structures. Acta Crystallogr. D.

[18] Zheng H., Chordia M.D., Cooper D.R., Chruszcz M., Muller P., Sheldrick G.M., Minor W. (2014). Validation of metal-binding sites in macromolecular structures with the CheckMyMetal web server. Nat. Protoc..

[19] Nayal M., Di Cera E. (1996). Valence screening of water in protein crystals reveals potential na+ binding sites. J. Mol. Biol..

[20] Zheng H., Chruszcz M., Lasota P., Lebioda L., Minor W. (2008). Data mining of metal ion environments present in protein structures. J. Inorg. Biochem..

[21] Allen F.H. (2002). The Cambridge structural database: a quarter of a million crystal structures and rising. Acta Cryst. B.

[22] Permyakov E. (2009). Metalloproteomics.

[23] Word J.M., Lovell S.C., LaBean T.H., Taylor H.C., Zalis M.E., Presley B.K., Richardson J.S., Richardson D.C. (1999). Visualizing and quantifying molecular goodness-of-fit: small-probe contact dots with explicit hydrogen atoms. J. Mol. Biol..

[24] Chen V.B., Arendall W.B. III, Headd J.J., Keedy D.A., Immormino R.M., Kapral G.J., Murray L.W., Richardson J.S., Richardson D.C. (2010). MolProbity: all-atom structure validation for macromolecular crystallography. Acta Crystallogr. D.

[25] Winn M.D., Ballard C.C., Cowtan K.D., Dodson E.J., Emsley P., Evans P.R., Keegan R.M., Krissinel E.B., Leslie A.G., McCoy A. (2011). Overview of the CCP4 suite and current developments. Acta Crystallogr. D Biol. Crystallogr..

[26] Brese N.E., O'Keeffe M. (1991). Bond-valence parameters for solids. Acta Cryst. B.

[27] Müller P., Köpke S., Sheldrick G.M. (2003). Is the bond-valence method able to identify metal atoms in protein structures. Acta Cryst. D.

[28] Borovinskaya M.A., Pai R.D., Zhang W., Schuwirth B.S., Holton J.M., Hirokawa G., Kaji H., Kaji A., Cate J.H. (2007). Structural basis for aminoglycoside inhibition of bacterial ribosome recycling. Nat. Struct. Mol. Biol..

[29] Rabl J., Leibundgut M., Ataide S.F., Haag A., Ban N. (2011). Crystal structure of the eukaryotic 40S ribosomal subunit in complex with initiation factor 1. Science.

[30] Petrov A.S., Bowman J.C., Harvey S.C., Williams L.D. (2011). Bidentate RNA-magnesium clamps: on the origin of the special role of magnesium in RNA folding. RNA.

[31] Szabó Z. (2008). Multinuclear NMR studies of the interaction of metal ions with adenine-nucleotides. Coord. Chem. Rev..

[32] Hawkins P.C., Warren G.L., Skillman A.G., Nicholls A. (2008). How to do an evaluation: pitfalls and traps. J. Comput. Aided Mol. Des..

[33] Andreini C., Cavallaro G., Lorenzini S., Rosato A. (2013). MetalPDB: a database of metal sites in biological macromolecular structures. Nucleic Acids Res..

[34] Castagnetto J.M., Hennessy S.W., Roberts V.A., Getzoff E.D., Tainer J.A., Pique M.E. (2002). MDB: the metalloprotein database and browser at the scripps research institute. Nucleic Acids Res..

[35] Hsin K., Sheng Y., Harding M.M., Taylor P., Walkinshaw M.D. (2008). MESPEUS: a database of the geometry of metal sites in proteins. J. Appl. Crystallogr..

[36] Philips A., Milanowska K., Lach G., Boniecki M., Rother K., Bujnicki J.M. (2012). MetalionRNA: computational predictor of metal-binding sites in RNA structures. Bioinformatics.

[37] Hanson R.M. (2010). Jmol – a paradigm shift in crystallographic visualization. J. Appl. Crystallogr..

