# Supplementary material for: Magnesium-binding architectures in RNA crystal structures: validation, binding preferences, classification and motif detection
Source: Nucleic Acids Res. 2015 Mar 23;43(7):3789–801. doi: 10.1093/nar/gkv225 (PMC4402538; doi:10.1093/nar/gkv225)
Supplement: SUPPLEMENTARY DATA [file supp_43_7_3789__index.html]

Magnesium-binding architectures in RNA crystal structures: validation, binding preferences, classification and motif detection — Magnesium-binding architectures in RNA crystal structures: validation, binding preferences, classification and motif detection — SUPPLEMENTARY DATA 

# Magnesium-binding architectures in RNA crystal structures: validation, binding preferences, classification and motif detection

## SUPPLEMENTARY DATA

**Files in this Data Supplement:**

- SUPPLEMENTARY DATA
