# Supplementary material for: Magnesium-binding architectures in RNA crystal structures: validation, binding preferences, classification and motif detection
Source: Nucleic Acids Res. 2015 Mar 23;43(7):3789–801. doi: 10.1093/nar/gkv225 (PMC4402538; doi:10.1093/nar/gkv225)

**SUPPLEMENTARY DATA**

**Supplementary Lexicon 1**

| inner-sphere (ligand) atom | An atom that forms a coordination bond with Mg^2+^. Each Mg^2+^ should have six inner-sphere atoms. |
| --- | --- |
| inner sphere | The ensemble of all inner-sphere atoms. |
| outer-sphere (ligand) atom | An atom that forms a hydrogen bond with an Mg^2+^ inner-sphere water. |
| outer sphere | The ensemble of all outer-sphere atoms or moieties. |
| moiety | A functional group of a nucleotide residue. Three distinct moieites are defined within a nucleotide residue: phosphate, ribose, and nucleobase. |
| ligand | A molecule (or its functional group) that is bound to Mg^2+^ through its inner or outer sphere. |
| Mg^2+^ site | Mg^2+^ atom in RNA strcuture together with all ligand atoms in its binding environment. |
| CN | Coordination number, defined as the number of inner-sphere atoms. |
| *V_i_* | Bond valence value derived from Mg^2+^-ligand distance of coordinating ligand *i.* |
| **v**_i_ | Bond valence vector of coordinating ligand *i*. |
| *Q_v_* | Quality parameter of valence defined as *Q_v_=* $1-\frac{\vert\sum V_{i}-2\vert}{2}$ , in which 2 is the oxidation state of Mg^2+^. |
| *Q_s_* | Quality parameter describing symmetrical distribution of the inner-sphere ligands around the Mg^2+^ defined as *Q_s_=*$1-\frac{\vert\text{v}_{1}+\text{v}_{2}+\ldots+\text{v}_{n}\vert}{\sum V_{i}}$*.* |
| *Q_e_* | Quality parameter describing the agreement of Mg^2+^ and the surrounding atoms B-factors defined as *Q_e_*=$\min\left( O_{m}, O_{e} \right)\times min(\frac{\frac{B_{m}}{O_{m}}}{\frac{B_{e}}{O_{e}}},\frac{\frac{B_{e}}{O_{e}}}{\frac{B_{m}}{O_{m}}})$ $\min\left( O_{m}, O_{e} \right)\times min(\frac{\frac{B_{m}}{O_{m}}}{\frac{B_{e}}{O_{e}}},\frac{\frac{B_{e}}{O_{e}}}{\frac{B_{m}}{O_{m}}})$ $\min\left( O_{m}, O_{e} \right)\times min(\frac{\frac{B_{m}}{O_{m}}}{\frac{B_{e}}{O_{e}}},\frac{\frac{B_{e}}{O_{e}}}{\frac{B_{m}}{O_{m}}})$.  *B_m_* the isotropic atomic displacement parameter (B-factor) of the Mg^2+^  *B_e_* the isotropic atomic displacement parameter of the Mg^2+^ environment  *O_m_* the occupancy of the Mg^2+^  *O_e_* the occupancy of the Mg^2+^ environment within 4 Å |
| full dataset | The dataset of all Mg^2+^ sites in RNA structures as of September 30, 2014. |
| benchmark dataset | The dataset of Mg^2+^ sites with sufficient quality extracted from the full dataset. See results section for the complete definition. |
| *F_atom_* | The frequency that a type of atom is observed to coordinate Mg^2+^ normalized by the frequency of that type of atom in the full dataset. |
| O_ph_ | RNA phosphate oxygen (OP1/OP2). |
| O_r_ | RNA ribose oxygen (O2’/O4’) or oxygen bridging phosphate and ribose (O3’/O5’). |
| O_b_ | RNA nucleobase oxygen. |
| N_b_ | RNA nucleobase nitrogen. |
| *cis-* | The two inner-sphere O_ph_ atoms form a O_ph_-Mg^2+^-O_ph_ angle of ~90°. |
| *trans-* | The two inner-sphere O_ph_ atoms form a O_ph_-Mg^2+^-O_ph_ angle of ~180°. |
| *fac-* | The three inner-sphere O_ph_ atoms form three O_ph_-Mg^2+^- O_ph_ angles of ~90° each. |
| *mer-* | The three inner-sphere O_ph_ atoms form two O_ph_-Mg^2+^- O_ph_ angles of ~90° each, and one O_ph_-Mg^2+^- O_ph_ angle of ~180°. |
| P_out_ | Outer-sphere phosphate moiety. |
| R_out_ | Outer-sphere ribose moiety. |
| B_out_ | Outer-sphere nucleobase moiety. |
| RNA-inner | Class of Mg^2+^ sites with only RNA atoms as non-water ligands in the inner sphere. |
| RNA-outer | Class of Mg^2+^ sites with only water molecules in the inner sphere and at least one RNA moiety in the outer sphere. |
| RNA-bound water | Water molecules that form hydrogen bonds with RNA atoms. |
| RNA-free water | Water molecules which do not form direct hydrogen bonds with RNA atoms. |

**Supplementary Text 1**

**Determination of thresholds for the validation parameters *Q_v_*, *Q_s_* and *Q_e_***

The threshold values for the validation parameters used to select the benchmark dataset were derived by examination of the distribution of each parameter for sites within the full dataset with CN=4-6 (28803 sites).

The *Q_v_* distribution shows two major peaks corresponding to sites with CN=6: one at *Q_v_*=0.97, which corresponds to sites with near the ideal Mg^2+^-O distance of 2.08 Å, and another at *Q_v_*=0.75 which corresponds mostly to sites with an Mg^2+^-water distance near 2.18 Å (Supplementary Figure S2). 2.18 Å is the default value in the library used by the refinement programs *REFMAC* (38) and *PHENIX* (39). The *Q_v_* minimum threshold value chosen was 0.5 (Supplementary Figure S3A), corresponding to Mg^2+^-O bond distances between 1.92 and 2.33 Å (for a typical Mg^2+^ site with CN=6 and Mg^2+^-O bond distances all equal).

Mg^2+^ sites with complete octahedral geometry (CN=6) and those missing 2 ligands (CN=4) in *trans*- conformation correspond to the highest peak on the *Q_s_* distribution plot (Supplementary Figure S3B). The other two peaks are caused by Mg^2+^ sites with one missing ligand (CN=5) or two missing ligands in a *cis-* conformation (CN=4). The *Q_s_* minimum threshold value was chosen at 0.6 to include all three peaks.

The *Q_e_* distribution exhibits a sharp peak at 1 and decreases gradually with lower *Q_e_* values (Supplementary Figure S3C). The *Q_e_* minimum threshold value chosen was 0.5, which indicates less than two-fold deviation between the B-factor of the metal and the average B-factor of the environment for sites with full occupancy.

Each of the selected threshold values are relatively permissive, allowing for the inclusion of as many reasonable sites as possible. However, taken together they filter out a number of highly questionable sites, which was further verified by visual inspection of hundreds of sites.

**Supplementary text 2**

**Magnesium ion binding preferences**

Statistical analysis of Mg^2+^ interactions within the benchmark dataset identified O_ph_ atoms and O_b_ atoms located on the edge of the nucleobase opposite to the sugar as the most predominant ligands for Mg^2+^ binding in inner sphere. Besides the most frequently observed phosphate oxygens (O_ph_) that provide charge neutralization, the frequencies for most other RNA oxygen atoms to serve as inner-sphere ligands can be explained in terms of steric accessibility. The O4’ atom of ribose (with the lowest *F_atom_*) is sterically occluded by other ribose, nucleobase, and sometimes even phosphate atoms in most RNA conformations. O3’ and O5’ are often occluded by adjacent ribose and phosphate atoms that limit accommodation of hexa-coordinated Mg^2+^. The two free electron pairs of the nucleobase O_b_ atoms adjacent to the ribose bond (C-O2 and U-O2) are rarely easily accessible. The electron pair directed towards ribose is blocked by steric clashes between Mg^2+^ inner-sphere waters and the ribose; while the other electron pair is usually blocked due to base pairing (40).

Overall, nucleobase nitrogens (N_b_) are less likely to serve as inner-sphere ligands. Among all 15 types of N_B_ atoms, only endocyclic nitrogen with a lone electron pair in the plane of the aromatic ring (–N=) atoms coordinated Mg^2+^ in the inner sphere with non-negligible frequency (even in the full dataset), and only the N7 atoms of adenine and guanine did so significantly (Figure 3, Supplementary Table S2). The other –N= atoms are not readily accessible due to steric occlusion: A-N3 and G-N3 are often blocked by the adjacent ribose moiety, while C-N3 and A-N1 are typically blocked by surrounding exocyclic groups (Figure 3). Nucleobase nitrogen atoms other than –N= (-NH-, -N<, -NH_2_) are poorly suited to coordinate Mg^2+^ directly. The only lone electron pair in –NH– and –N< is delocalized and directed perpendicular to the aromatic ring, which is unfavorable for Mg^2+^ coordination. Exocyclic amino groups (–NH_2_) delocalize their lone electron pair into the heterocyclic ring and can coordinate metals only after deprotonation or proton tautomeric shift, which can be induced by transition metals like platinum or zinc, but not by Mg^2+^ (11). These considerations allowed us to implement a rule used during the search for inner-sphere atoms: namely that the only allowed coordinating nitrogen from a nucleobase is an endocyclic nitrogen with a lone electron pair in the plane of the aromatic ring (–N=). Moreover we excluded sites which have nitrogen atoms other than –N= (meaning -NH-, -N<, -NH_2_) in the inner-sphere from the benchmark dataset as chemically unfavorable.

In the case of outer-sphere coordination, the RNA moiety is more distant from Mg^2+^, which diminishes the effect of steric occlusion.Therefore outer-sphere hydrogen bonds are less sensitive to steric accessibility; e.g. ribose oxygens O3’ and O5’ are common outer-sphere ligands. Moreover, most nitrogen atoms are fully capable to form outer-sphere hydrogen bonds unless it has been involved in base pairing, especially nucleobase exocyclic amino groups (-NH_2_).

**References**

38. Murshudov,G.N., Skubak,P., Lebedev,A.A., Pannu,N.S., Steiner,R.A., Nicholls,R.A., Winn,M.D., Long,F. and Vagin,A.A. (2011) REFMAC5 for the refinement of macromolecular crystal structures. *Acta Crystallogr. D,* **67,** 355-367.

39. Adams,P.D., Afonine,P.V., Bunkoczi,G., Chen,V.B., Davis,I.W., Echols,N., Headd,J.J., Hung,L.W., Kapral,G.J., Grosse-Kunstleve,R.W., et al. (2010) PHENIX: A comprehensive python-based system for macromolecular structure solution. *Acta Crystallogr. D,* **66,** 213-221.

40. Xin,Y. and Olson,W.K. (2009) BPS: A database of RNA base-pair structures. *Nucleic Acids Res.,* **37,** D83-8.

**SUPPLEMENTARY TABLES AND FIGURES**

Table S1. Mg^2+^ distance-to-valence conversion. The distance is shown along with corresponding valence in the first two columns for both Mg-O and Mg-N. Due to the typical octahedral geometry of Mg^2+^ having six ligands, six times of the bond valence value is also shown for convenience purpose.

Table S2. Normalized frequencies of RNA atoms for Mg^2+^ coordination in the inner and outer sphere. The number of atoms of each type within the full dataset (the last column) was used to normalize interaction frequencies. The atom types with the highest *F_atom_* values are highlighted in green.

Table S3. Structural classification of RNA-inner Mg^2+^ sites into 14 subclasses and 41 types. Site types are represented by a code listing the characteristic inner-sphere composition. The geometric isomer prefixes *cis-/trans-/fac-/mer-* are used in the code to define the relative position of inner-sphere O_P_ atoms around the Mg^2+^.

Table S4. Structural classification of RNA-outer Mg^2+^ sites into 39 subclasses and 95 types. Site types are represented by a code listing the characteristic outer-sphere composition.

Figure S1. Percentages of Mg^2+^ sites from ribosome (red bar) and non-ribosome structures (blue bar) in different resolution ranges. The total number of Mg^2+^ sites for each resolution bin is shown on top of each bar.

Figure S2. (A) Distribution of Mg^2+^-O distances for Mg^2+^ sites with *Q_v_*>=0.50 exhibit a bimodal distribution with peak distances at around 2.08 Ǻ and 2.18 Ǻ. (B) The distribution of Mg^2+^-O distances for Mg^2+^ sites with 0.72<=*Q_v_*<=0.77 exhibit a major peak distance at around 2.18 Ǻ. The distance distribution is binned every 0.02 Ǻ.

Figure S3. Distribution of quality parameters *Q_v_* (A), *Q_s_* (B) and *Q_e_* (C) for Mg^2+^ sites with CN=4-6 within the full dataset. For each parameter, the minimum thresholds used to define validated sites are indicated by red lines and labeled. The most prominent peaks are labeled as described in the Supplementary Text 1 with green CN labels referring to optimal geometries and blue labels to sub-optimal geometries.

Figure S4. Distance distribution of (A) Mg^2+^-water (B) Mg^2+^-O (non-water) and (C) Mg^2+^-N interactions within the inner sphere for benchmark and non-benchmark Mg^2+^ sites.

Figure S5. Representative sites of three RNA-inner and three RNA-outer validated Mg^2+^ binding motifs previously reported in the literature according to Table 2. The figure was prepared in the same style and manner as in Figure 6 in the main text. For each representative site, PDB code with Mg2+ residue number is provided with structure resolution and Q-values in parenthesis (Qv / Qs / Qe). (I) 3OWW-A103, 2.8 Å (0.6 / 1.0 / 0.7); (II) 3DD2-B1000, 1.9 Å (0.9 / 0.9 / 1.0); (III) 1VQ8-08004, 2.2 Å (1.0 / 0.9 / 0.9); (IV) 4TP8-A1601, 2.8 Å (0.7 / 1.0 / 0.9); (V) 3HAX-E200, 2.11 Å (0.7 / 1.0 / 1.0); (VI) 2QBC-B3337, 3.54 Å (1.0 / 1.0 / 1.0).Table S1.

| Mg-O | valence | valence×6 |  | Mg-N | valence | valence×6 |
| --- | --- | --- | --- | --- | --- | --- |
| 1.60 | 1.21 | 7.25 |  | 1.60 | 1.72 | 10.30 |
| 1.62 | 1.14 | 6.87 |  | 1.62 | 1.63 | 9.76 |
| 1.64 | 1.08 | 6.51 |  | 1.64 | 1.54 | 9.25 |
| 1.66 | 1.03 | 6.16 |  | 1.66 | 1.46 | 8.76 |
| 1.68 | 0.97 | 5.84 |  | 1.68 | 1.38 | 8.30 |
| 1.70 | 0.92 | 5.53 |  | 1.70 | 1.31 | 7.86 |
| 1.72 | 0.87 | 5.24 |  | 1.72 | 1.24 | 7.45 |
| 1.74 | 0.83 | 4.97 |  | 1.74 | 1.18 | 7.06 |
| 1.76 | 0.78 | 4.70 |  | 1.76 | 1.11 | 6.69 |
| 1.78 | 0.74 | 4.46 |  | 1.78 | 1.06 | 6.33 |
| 1.80 | 0.70 | 4.22 |  | 1.80 | 1.00 | 6.00 |
| 1.82 | 0.67 | 4.00 |  | 1.82 | 0.95 | 5.68 |
| 1.84 | 0.63 | 3.79 |  | 1.84 | 0.90 | 5.39 |
| 1.86 | 0.60 | 3.59 |  | 1.86 | 0.85 | 5.10 |
| 1.88 | 0.57 | 3.40 |  | 1.88 | 0.81 | 4.83 |
| 1.90 | 0.54 | 3.22 |  | 1.90 | 0.76 | 4.58 |
| 1.92 | 0.51 | 3.05 |  | 1.92 | 0.72 | 4.34 |
| 1.94 | 0.48 | 2.89 |  | 1.94 | 0.68 | 4.11 |
| 1.96 | 0.46 | 2.74 |  | 1.96 | 0.65 | 3.89 |
| 1.98 | 0.43 | 2.60 |  | 1.98 | 0.61 | 3.69 |
| 2.00 | 0.41 | 2.46 |  | 2.00 | 0.58 | 3.49 |
| 2.02 | 0.39 | 2.33 |  | 2.02 | 0.55 | 3.31 |
| 2.04 | 0.37 | 2.21 |  | 2.04 | 0.52 | 3.14 |
| 2.06 | 0.35 | 2.09 |  | 2.06 | 0.50 | 2.97 |
| 2.08 | 0.33 | 1.98 |  | 2.08 | 0.47 | 2.82 |
| 2.10 | 0.31 | 1.88 |  | 2.10 | 0.44 | 2.67 |
| 2.12 | 0.30 | 1.78 |  | 2.12 | 0.42 | 2.53 |
| 2.14 | 0.28 | 1.68 |  | 2.14 | 0.40 | 2.39 |
| 2.16 | 0.27 | 1.60 |  | 2.16 | 0.38 | 2.27 |
| 2.18 | 0.25 | 1.51 |  | 2.18 | 0.36 | 2.15 |
| 2.20 | 0.24 | 1.43 |  | 2.20 | 0.34 | 2.04 |
| 2.22 | 0.23 | 1.36 |  | 2.22 | 0.32 | 1.93 |
| 2.24 | 0.21 | 1.29 |  | 2.24 | 0.30 | 1.83 |
| 2.26 | 0.20 | 1.22 |  | 2.26 | 0.29 | 1.73 |
| 2.28 | 0.19 | 1.15 |  | 2.28 | 0.27 | 1.64 |
| 2.30 | 0.18 | 1.09 |  | 2.30 | 0.26 | 1.55 |
| 2.32 | 0.17 | 1.04 |  | 2.32 | 0.25 | 1.47 |
| 2.34 | 0.16 | 0.98 |  | 2.34 | 0.23 | 1.39 |
| 2.36 | 0.15 | 0.93 |  | 2.36 | 0.22 | 1.32 |
| 2.38 | 0.15 | 0.88 |  | 2.38 | 0.21 | 1.25 |

| Table S1 (continued). | | | | | | |
| --- | --- | --- | --- | --- | --- | --- |
| Mg-O | valence | 6*valence |  | Mg-N | valence | 6*valence |
| 2.40 | 0.14 | 0.83 |  | 2.40 | 0.20 | 1.19 |
| 2.42 | 0.13 | 0.79 |  | 2.42 | 0.19 | 1.12 |
| 2.44 | 0.12 | 0.75 |  | 2.44 | 0.18 | 1.06 |
| 2.46 | 0.12 | 0.71 |  | 2.46 | 0.17 | 1.01 |
| 2.48 | 0.11 | 0.67 |  | 2.48 | 0.16 | 0.95 |
| 2.50 | 0.11 | 0.64 |  | 2.50 | 0.15 | 0.90 |
| 2.52 | 0.10 | 0.60 |  | 2.52 | 0.14 | 0.86 |
| 2.54 | 0.10 | 0.57 |  | 2.54 | 0.14 | 0.81 |
| 2.56 | 0.09 | 0.54 |  | 2.56 | 0.13 | 0.77 |
| 2.58 | 0.09 | 0.51 |  | 2.58 | 0.12 | 0.73 |
| 2.60 | 0.08 | 0.49 |  | 2.60 | 0.12 | 0.69 |
| 2.62 | 0.08 | 0.46 |  | 2.62 | 0.11 | 0.65 |
| 2.64 | 0.07 | 0.44 |  | 2.64 | 0.10 | 0.62 |
| 2.66 | 0.07 | 0.41 |  | 2.66 | 0.10 | 0.59 |
| 2.68 | 0.07 | 0.39 |  | 2.68 | 0.09 | 0.56 |
| 2.70 | 0.06 | 0.37 |  | 2.70 | 0.09 | 0.53 |
| 2.72 | 0.06 | 0.35 |  | 2.72 | 0.08 | 0.50 |
| 2.74 | 0.06 | 0.33 |  | 2.74 | 0.08 | 0.47 |
| 2.76 | 0.05 | 0.32 |  | 2.76 | 0.07 | 0.45 |
| 2.78 | 0.05 | 0.30 |  | 2.78 | 0.07 | 0.42 |
| 2.80 | 0.05 | 0.28 |  | 2.80 | 0.07 | 0.40 |
| 2.82 | 0.04 | 0.27 |  | 2.82 | 0.06 | 0.38 |
| 2.84 | 0.04 | 0.25 |  | 2.84 | 0.06 | 0.36 |
| 2.86 | 0.04 | 0.24 |  | 2.86 | 0.06 | 0.34 |
| 2.88 | 0.04 | 0.23 |  | 2.88 | 0.05 | 0.32 |
| 2.90 | 0.04 | 0.22 |  | 2.90 | 0.05 | 0.31 |
| 2.92 | 0.03 | 0.20 |  | 2.92 | 0.05 | 0.29 |
| 2.94 | 0.03 | 0.19 |  | 2.94 | 0.05 | 0.28 |
| 2.96 | 0.03 | 0.18 |  | 2.96 | 0.04 | 0.26 |
| 2.98 | 0.03 | 0.17 |  | 2.98 | 0.04 | 0.25 |
| 3.00 | 0.03 | 0.16 |  | 3.00 | 0.04 | 0.23 |
| 3.02 | 0.03 | 0.16 |  | 3.02 | 0.04 | 0.22 |
| 3.04 | 0.02 | 0.15 |  | 3.04 | 0.04 | 0.21 |
| 3.06 | 0.02 | 0.14 |  | 3.06 | 0.03 | 0.20 |
| 3.08 | 0.02 | 0.13 |  | 3.08 | 0.03 | 0.19 |
| 3.10 | 0.02 | 0.13 |  | 3.10 | 0.03 | 0.18 |
| 3.12 | 0.02 | 0.12 |  | 3.12 | 0.03 | 0.17 |
| 3.14 | 0.02 | 0.11 |  | 3.14 | 0.03 | 0.16 |
| 3.16 | 0.02 | 0.11 |  | 3.16 | 0.03 | 0.15 |
| 3.18 | 0.02 | 0.10 |  | 3.18 | 0.02 | 0.14 |
| 3.20 | 0.02 | 0.10 |  | 3.20 | 0.02 | 0.14 |

Table S2.

| Atom Name | | Inner-sphere interactions | | | | Outer-sphere interactions | | | | Number of atoms in the full dataset |
| --- | --- | --- | --- | --- | --- | --- | --- | --- | --- | --- |
|  |  | *F_atom_* | Number of interactions in the benchmark dataset | Number of interactions in the full dataset | Percentage of inetractions retained in the benchmark dataset | *F_atom_* | Number of interactions in the benchmark dataset | Number of interactions in the full dataset | Percentage of inetractions retained in the bench-mark dataset |  |
| O_ph_ | OP1 | 4.19 | 6240 | 26844 | 23.3 | 1.91 | 12178 | 21224 | 57.4 | 1211834 |
|  | OP2 | 4.99 | 7428 | 31952 | 23.3 | 2.78 | 17769 | 30291 | 58.7 | 1211834 |
| O_r_ | O2' | 0.07 | 101 | 4801 | 2.10 | 0.54 | 3455 | 6630 | 52.1 | 1211834 |
|  | O3' | 0.04 | 53 | 2341 | 2.26 | 0.55 | 3497 | 6008 | 58.2 | 1211834 |
|  | O4' | 0.004 | 6 | 884 | 0.68 | 0.07 | 449 | 797 | 56.3 | 1211834 |
|  | O5' | 0.04 | 58 | 948 | 6.12 | 0.61 | 3916 | 6068 | 64.5 | 1211834 |
| A | N1 (-N=) | 0.04 | 15 | 330 | 4.55 | 0.65 | 982 | 1464 | 67.1 | 285184 |
|  | N3 (-N=) | 0.003 | 1 | 424 | 0.24 | 0.20 | 294 | 749 | 39.3 | 285184 |
|  | N6 (NH_2_) | - | 0 | 60 | 0 | 0.98 | 1476 | 2541 | 58.1 | 285184 |
|  | N7 (-N=) | 0.74 | 258 | 1386 | 18.6 | 2.44 | 3674 | 5387 | 68.2 | 285184 |
|  | N9 (R-N<) | 0 | 0 | 13 | 0 | 0.0007 | 1 | 19 | 5.3 | 285184 |
| G | O6 (sugar opposite) | 1.45 | 719 | 13258 | 5.42 | 3.84 | 8169 | 15462 | 52.8 | 403628 |
|  | N1 (-NH-) | 0 | 0 | 102 | 0 | 0.22 | 472 | 780 | 60.5 | 403628 |
|  | N2 (NH_2_) | 0 | 0 | 110 | 0 | 0.11 | 235 | 396 | 59.3 | 403628 |
|  | N3 (-N=) | 0.002 | 1 | 295 | 0.34 | 0.12 | 260 | 544 | 47.8 | 403628 |
|  | N7 (-N=) | 1.35 | 669 | 4769 | 14.0 | 3.62 | 7694 | 13822 | 55.7 | 403628 |
|  | N9 (R-N<) | 0 | 0 | 23 | 0 | 0.01 | 23 | 36 | 63.9 | 403628 |
| C | O2 (sugar side) | 0.14 | 53 | 1555 | 3.41 | 0.36 | 585 | 1095 | 53.4 | 309202 |
|  | N1(R-N<) | 0 | 0 | 7 | 0 | 0.008 | 13 | 32 | 40.6 | 309202 |
|  | N3 (-N=) | 0.01 | 4 | 372 | 1.08 | 0.33 | 541 | 857 | 63.1 | 309202 |
|  | N4 (NH_2_) | 0 | 0 | 99 | 0 | 0.50 | 807 | 1324 | 61.0 | 309202 |
| U | O2 (sugar side) | 0.008 | 2 | 693 | 0.29 | 0.22 | 249 | 479 | 52.0 | 213820 |
|  | O4 (sugar opposite) | 2.33 | 612 | 6168 | 9.92 | 2.47 | 2789 | 4958 | 56.3 | 213820 |
|  | N1 (R-N<) | 0 | 0 | 10 | 0 | 0.003 | 3 | 6 | 50.0 | 213820 |
|  | N3 (-NH-) | 0 | 0 | 38 | 0 | 0.08 | 93 | 202 | 46.0 | 213820 |

Table S3.

| **Class** | **Subclass** | | **Type** | **Number of sites** |
| --- | --- | --- | --- | --- |
| **RNA-inner** | **Number of atoms in the inner-sphere** | |  |  |
|  | **O_ph_** | **O_r_\|O_b_\|N_b_** |  |  |
|  | **#O_ph_=0**  **(987)** | **#(O_r_\|O_b_\|N_b_)=1** | **O_r_** | **22** |
|  |  |  | **O_b_** | **468** |
|  |  |  | **N_b_** | **284** |
|  |  | **#(O_r_\|O_b_\|N_b_)=2** | **2O_r_** | **6** |
|  |  |  | **O_r_•O_b_** | **1** |
|  |  |  | **2O_b_** | **16** |
|  |  |  | **O_b_•N_b_** | **26** |
|  |  |  | **2N_b_** | **158** |
|  |  | **#(O_r_\|O_b_\|N_b_)=3** | **2O_b_•N_b_** | **1** |
|  |  |  | **O_b_•2N_b_** | **3** |
|  |  |  | **2O_r_•N_b_** | **1** |
|  |  | **#(O_r_\|O_b_\|N_b_)=4** | **2O_r_•2O_b_** | **1** |
|  | **#O_ph_=1**  **(5379)** | **#(O_r_\|O_b_\|N_b_)=0** | **O_ph_** | **588** |
|  |  |  | **O_ph_•P_out_** | **1301** |
|  |  |  | **O_ph_•2P_out_** | **1607** |
|  |  |  | **O_ph_•3P_out_** | **867** |
|  |  |  | **O_ph_•4P_out_** | **231** |
|  |  |  | **O_ph_•5P_out_** | **37** |
|  |  | **#(O_r_\|O_b_\|N_b_)=1** | **O_ph_•O_r_** | **49** |
|  |  |  | **O_ph_•O_b_** | **554** |
|  |  |  | **O_ph_•N_b_** | **134** |
|  |  | **#(O_r_\|O_b_\|N_b_)=2** | **O_ph_•2O_r_** | **8** |
|  |  |  | **O_ph_•2O_b_** | **2** |
|  |  | **#(O_r_\|O_b_\|N_b_)=3** | **O_ph_•O_r_•2O_b_** | **1** |
|  | **#O_ph_=2**  **(2287)** | **#(O_r_\|O_b_\|N_b_)=0** | ***cis*-2O_ph_** | **1720** |
|  |  |  | ***trans*-2O_ph_** | **282** |
|  |  | **#(O_r_\|O_b_\|N_b_)=1** | ***cis*-2O_ph_•O_r_** | **36** |
|  |  |  | ***cis*-2O_ph_•O_b_** | **98** |
|  |  |  | ***cis*-2O_ph_•N_b_** | **118** |
|  |  | **#(O_r_\|O_b_\|N_b_)=2** | ***cis*-2O_ph_•2O_r_** | **2** |
|  |  |  | ***cis*-2O_ph_•O_r_•O_b_** | **2** |
|  |  |  | ***cis*-2O_ph_•2O_b_** | **28** |
|  |  |  | ***trans*-2O_ph_•2O_b_** | **1** |
|  | **#O_ph_=3**  **(911)** | **#(O_r_\|O_b_\|N_b_)=0** | ***fac*-3O_ph_** | **392** |
|  |  |  | ***mer*-3O_ph_** | **430** |
|  |  | **#(O_r_\|O_b_\|N_b_)=1** | ***fac*-3O_ph_•O_r_** | **3** |
|  |  |  | ***mer*-3O_ph_•O_r_** | **19** |
|  |  |  | ***fac*-3O_ph_•O_b_** | **64** |
|  |  |  | ***mer*-3O_ph_•O_b_** | **3** |
|  | **#O_ph_=4 (64)** | **#(O_r_\|O_b_\|N_b_)=0** | ***trans*,*cis*-4O_ph_** | **29** |
|  |  |  | ***trans*,*trans*-4O_ph_** | **35** |

Table S4.

| **Class** | **Subclass** | | **Type** | **Number of sites** |
| --- | --- | --- | --- | --- |
| **RNA-outer** | **Number of moieties in the outer-sphere** | |  |  |
|  | **Phosphate** | **Ribose\|Base** |  |  |
|  | **#P_out_=0**  **(1355)** | **#(R_out_\|B_out_)=1** | **R_out_** | **25** |
|  |  |  | **B_out_** | **132** |
|  |  | **#(R_out_\|B_out_)=2** | **2R_out_** | **1** |
|  |  |  | **R_out_•B_out_** | **12** |
|  |  |  | **2B_out_** | **555** |
|  |  | **#(R_out_\|B_out_)=3** | **2R_out_•B_out_** | **3** |
|  |  |  | **R_out_•2B_out_** | **12** |
|  |  |  | **3B_out_** | **341** |
|  |  | **#(R_out_\|B_out_)=4** | **2R_out_•2B_out_** | **5** |
|  |  |  | **R_out_•3B_out_** | **13** |
|  |  |  | **4B_out_** | **105** |
|  |  | **#(R_out_\|B_out_)=5** | **2R_out_•3B_out_** | **4** |
|  |  |  | **R_out_•4B_out_** | **5** |
|  |  |  | **5B_out_** | **45** |
|  |  | **#(R_out_\|B_out_)=6** | **4R_out_•2B_out_** | **1** |
|  |  |  | **2R_out_•4B_out_** | **1** |
|  |  |  | **R_out_•5B_out_** | **2** |
|  |  |  | **6B_out_** | **78** |
|  |  | **#(R_out_\|B_out_)=7** | **3R_out_•4B_out_** | **1** |
|  |  |  | **2R_out_•5B_out_** | **3** |
|  |  |  | **R_out_•6B_out_** | **2** |
|  |  |  | **7B_out_** | **7** |
|  |  | **#(R_out_\|B_out_)=8** | **4R_out_•4B_out_** | **1** |
|  |  |  | **R_out_•7B_out_** | **1** |
|  | **#P_out_=1**  **(1319)** | **#(R_out_\|B_out_)=0** | **P_out_** | **94** |
|  |  | **#(R_out_\|B_out_)=1** | **P_out_•R_out_** | **37** |
|  |  |  | **P_out_•B_out_** | **223** |
|  |  | **#(R_out_\|B_out_)=2** | **P_out_•2R_out_** | **3** |
|  |  |  | **P_out_•R_out_•B_out_** | **28** |
|  |  |  | **P_out_•2B_out_** | **343** |
|  |  | **#(R_out_\|B_out_)=3** | **P_out_•2R_out_•B_out_** | **3** |
|  |  |  | **P_out_•R_out_•2B_out_** | **40** |
|  |  |  | **P_out_•3B_out_** | **341** |
|  |  | **#(R_out_\|B_out_)=4** | **P_out_•3R_out_•B_out_** | **1** |
|  |  |  | **P_out_•2R_out_•2B_out_** | **4** |
|  |  |  | **P_out_•R_out_•3B_out_** | **32** |
|  |  |  | **P_out_• 4B_out_** | **135** |
|  |  | **#(R_out_\|B_out_)=5** | **P_out_•4R_out_•B_out_** | **1** |
|  |  |  | **P_out_•3R_out_•2B_out_** | **1** |
|  |  |  | **P_out_•2R_out_•3B_out_** | **5** |
|  |  |  | **P_out_•R_out_•4B_out_** | **6** |
|  |  |  | **P_out_•5B_out_** | **18** |
|  |  | **#(R_out_\|B_out_)=6** | **P_out_•6B_out_** | **4** |
|  | **#P_out_=2**  **(1103)** | **#(R_out_\|B_out_)=0** | **2P_out_** | **191** |
|  |  | **#(R_out_\|B_out_)=1** | **2P_out_•R_out_** | **39** |
|  |  |  | **2P_out_•B_out_** | **169** |
|  |  | **#(R_out_\|B_out_)=2** | **2P_out_•2R_out_** | **2** |
|  |  |  | **2P_out_•R_out_•B_out_** | **32** |
|  |  |  | **2P_out_•2B_out_** | **279** |
|  |  | **#(R_out_\|B_out_)=3** | **2P_out_•2R_out_•B_out_** | **3** |
|  |  |  | **2P_out_•R_out_•2B_out_** | **33** |
|  |  |  | **2P_out_•3B_out_** | **199** |
|  |  | **#(R_out_\|B_out_)=4** | **2P_out_•2R_out_•2B_out_** | **12** |
|  |  |  | **2P_out_•R_out_•3B_out_** | **39** |
|  |  |  | **2P_out_•4B_out_** | **68** |
|  |  | **#(R_out_\|B_out_)=5** | **2P_out_•3R_out_•2B_out_** | **1** |
|  |  |  | **2P_out_•2R_out_•3B_out_** | **11** |
|  |  |  | **2P_out_•R_out_•4B_out_** | **9** |
|  |  |  | **2P_out_•5B_out_** | **12** |
|  |  | **#(R_out_\|B_out_)=6** | **2P_out_•R_out_•5B_out_** | **1** |
|  |  |  | **2P_out_•6B_out_** | **3** |

Table S4 (continued).

| **RNA-outer** | **#P_out_=3**  **(783)** | **#(R_out_\|B_out_)=0** | **3P_out_** | **207** |
| --- | --- | --- | --- | --- |
|  |  | **#(R_out_\|B_out_)=1** | **3P_out_•R_out_** | **51** |
|  |  |  | **3P_out_•B_out_** | **155** |
|  |  | **#(R_out_\|B_out_)=2** | **3P_out_•2R_out_** | **11** |
|  |  |  | **3P_out_•R_out_•B_out_** | **36** |
|  |  |  | **3P_out_•2B_out_** | **131** |
|  |  | **#(R_out_\|B_out_)=3** | **3P_out_•3R_out_** | **1** |
|  |  |  | **3P_out_•2R_out_•B_out_** | **6** |
|  |  |  | **3P_out_•R_out_•2B_out_** | **58** |
|  |  |  | **3P_out_•3B_out_** | **34** |
|  |  | **#(R_out_\|B_out_)=4** | **3P_out_•2R_out_•2B_out_** | **4** |
|  |  |  | **3P_out_•R_out_•3B_out_** | **33** |
|  |  |  | **3P_out_•4B_out_** | **24** |
|  |  | **#(R_out_\|B_out_)=5** | **3P_out_•2R_out_•3B_out_** | **4** |
|  |  |  | **3P_out_•R_out_•4B_out_** | **16** |
|  |  |  | **3P_out_•5B_out_** | **10** |
|  |  | **#(R_out_\|B_out_)=6** | **3P_out_•R_out_•5B_out_** | **2** |
|  | **#P_out_=4**  **(412)** | **#(R_out_\|B_out_)=0** | **4P_out_** | **174** |
|  |  | **#(R_out_\|B_out_)=1** | **4P_out_•R_out_** | **51** |
|  |  |  | **4P_out_•B_out_** | **125** |
|  |  | **#(R_out_\|B_out_)=2** | **4P_out_•2R_out_** | **4** |
|  |  |  | **4P_out_•R_out_•B_out_** | **10** |
|  |  |  | **4P_out_•2B_out_** | **40** |
|  |  | **#(R_out_\|B_out_)=3** | **4P_out_•R_out_•2B_out_** | **3** |
|  |  |  | **4P_out_•3B_out_** | **2** |
|  |  | **#(R_out_\|B_out_)=4** | **4P_out_•R_out_•3B_out_** | **1** |
|  |  | **#(R_out_\|B_out_)=5** | **4P_out_•3R_out_•2B_out_** | **1** |
|  |  |  | **4P_out_•R_out_•4B_out_** | **1** |
|  | **#P_out_=5**  **(54)** | **#(R_out_\|B_out_)=0** | **5P_out_** | **20** |
|  |  | **#(R_out_\|B_out_)=1** | **5P_out_•R_out_** | **12** |
|  |  |  | **5P_out_•B_out_** | **6** |
|  |  | **#(R_out_\|B_out_)=2** | **5P_out_•R_out_•B_out_** | **5** |
|  |  |  | **5P_out_•2B_out_** | **10** |
|  |  | **#(R_out_\|B_out_)=3** | **5P_out_•3R_out_** | **1** |

Figure S1.


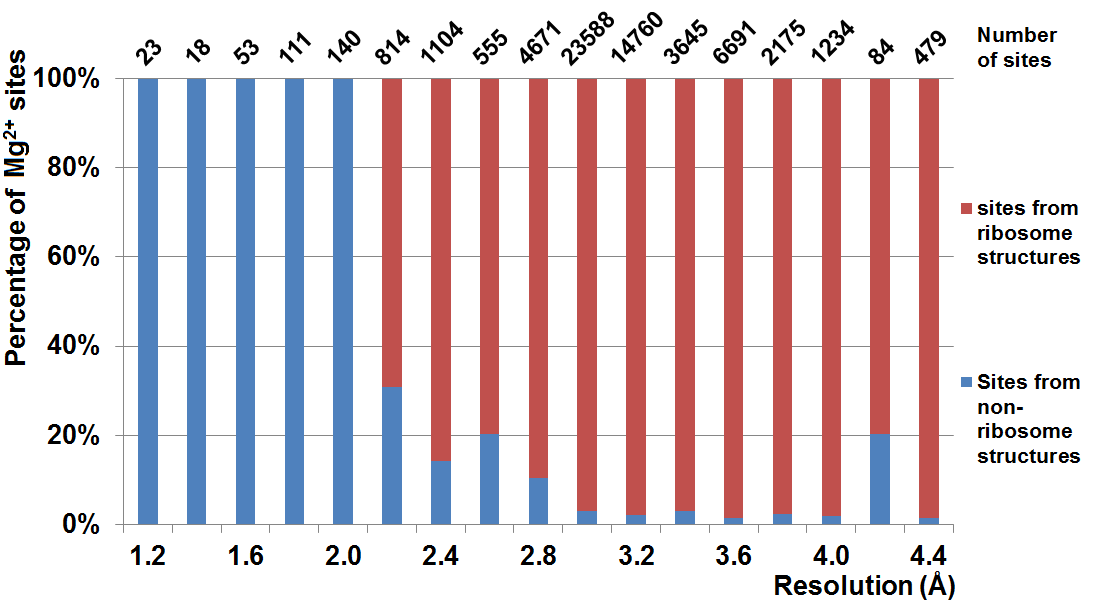


Figure S2.


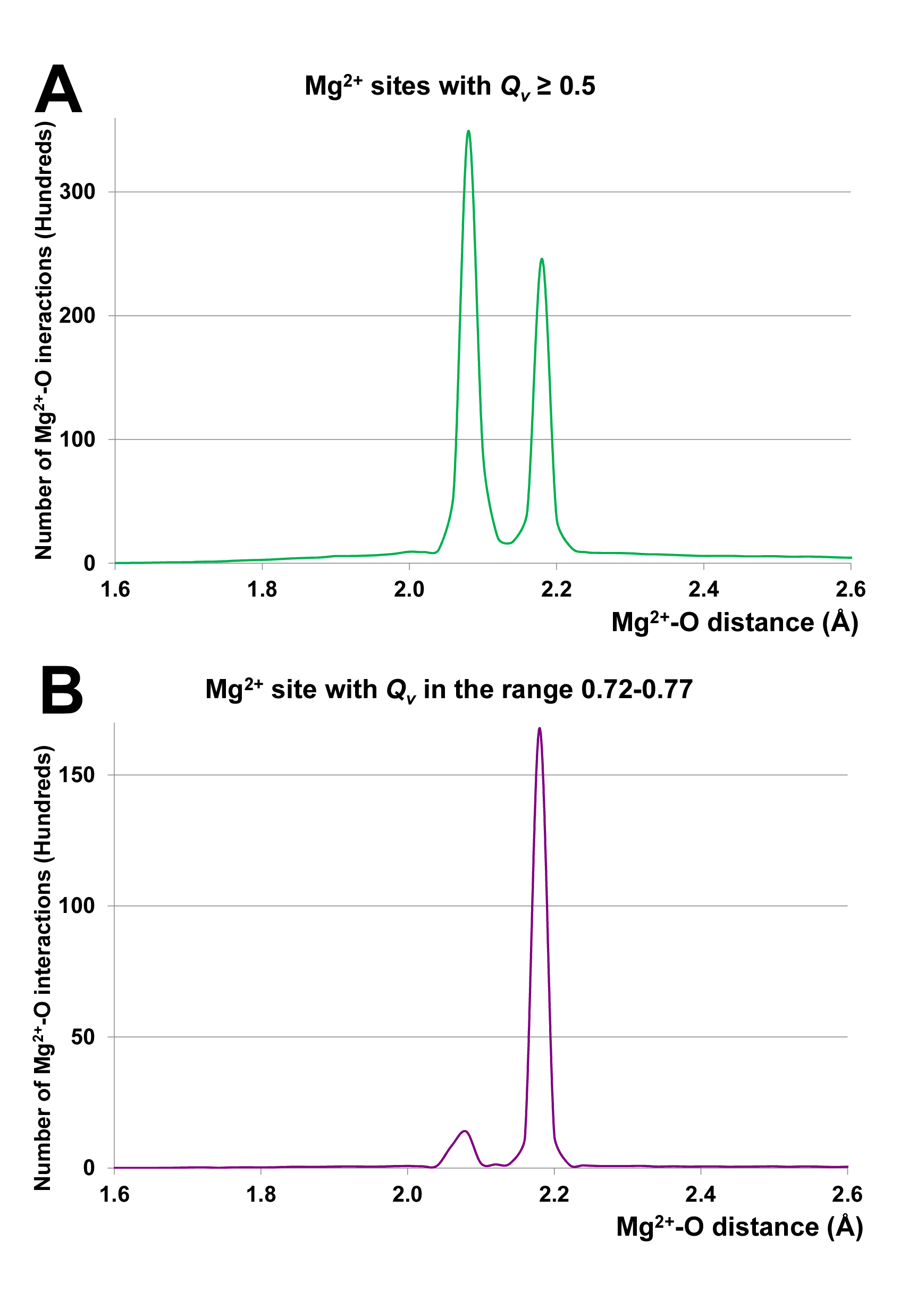


Figure S3.


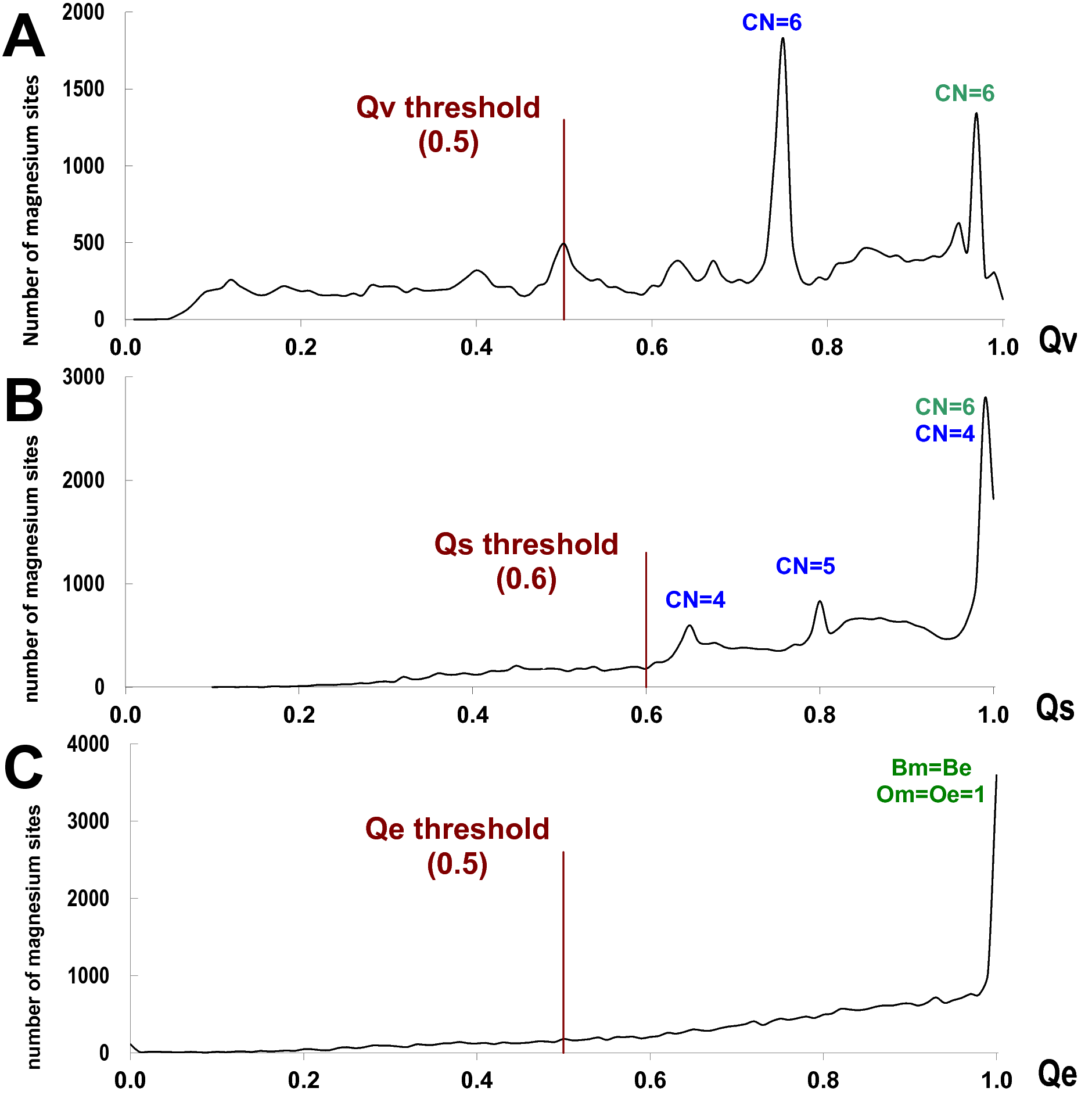
Figure S4.


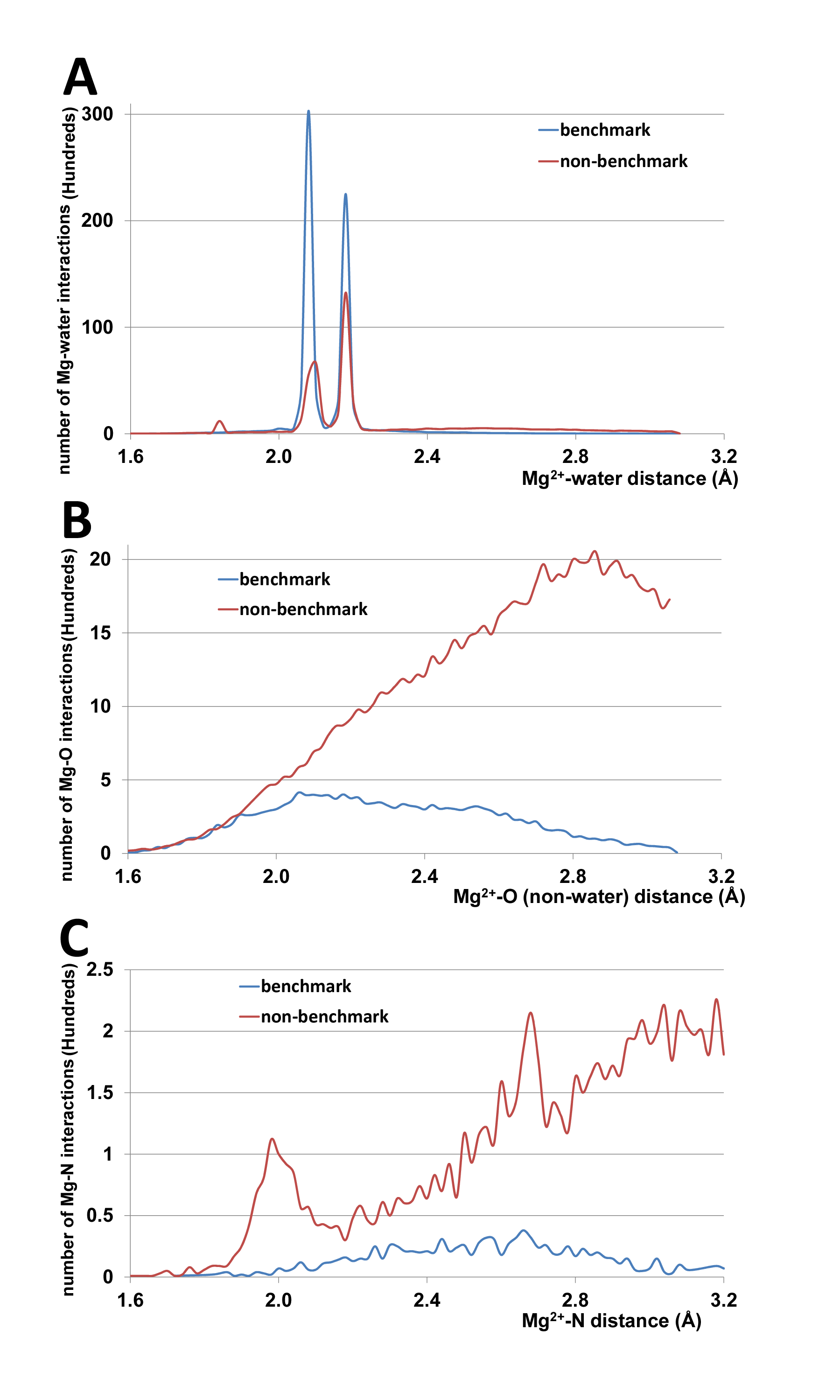


Figure S5


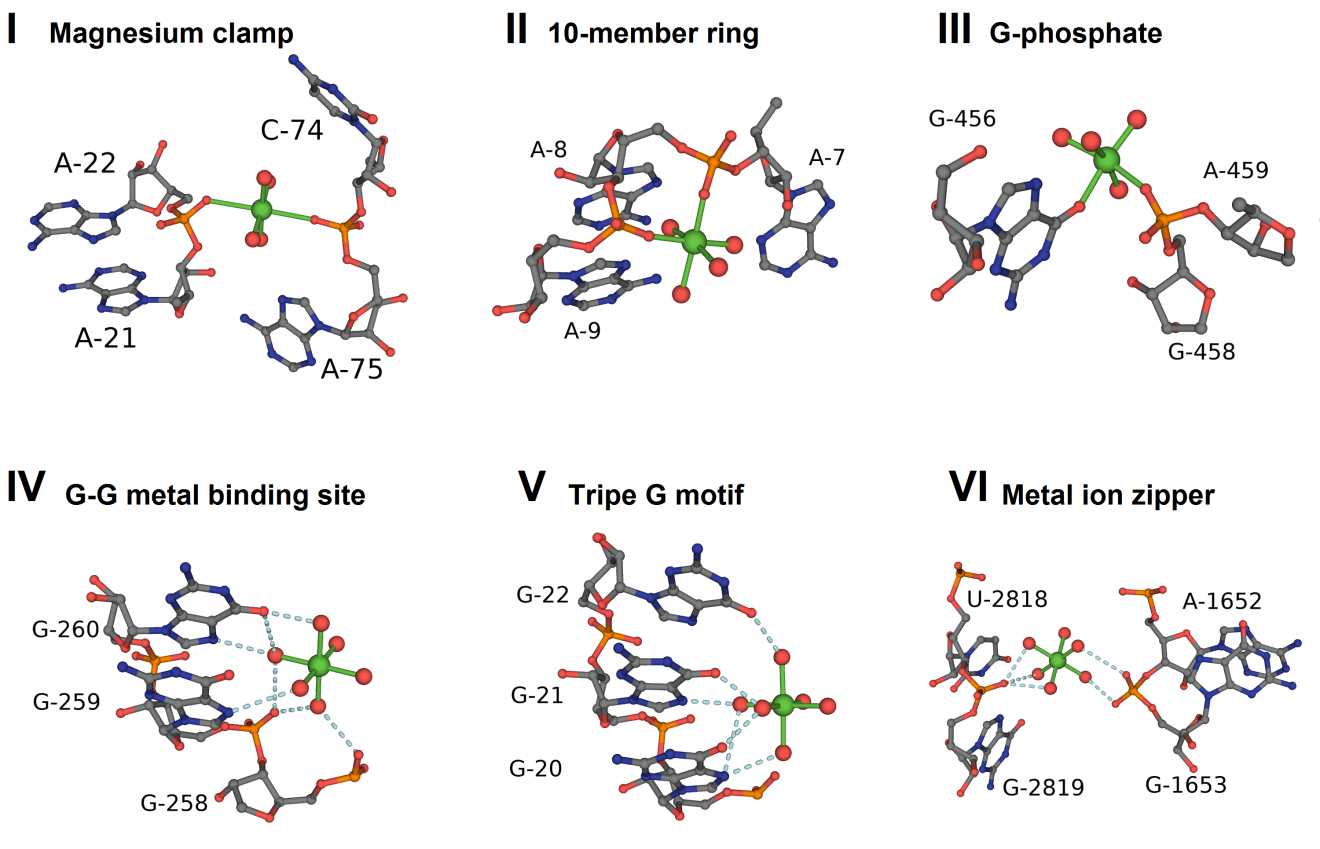

Supplement: SUPPLEMENTARY DATA [file supp_gkv225_nar-00017-r-2015-File008.docx]
